# Tandem Electroreduction of Nitrate to Ammonia Using a Cobalt–Copper Mixed Single‐Atom/Cluster Catalyst with Synergistic Effects

**DOI:** 10.1002/advs.202407250

**Published:** 2024-09-19

**Authors:** Jungwon Suh, Hyeonuk Choi, Yujin Kong, Jihun Oh

**Affiliations:** ^1^ Department of Materials Science and Engineering Korea Advanced Institute of Science and Technology (KAIST) 291 Daehak‐ro, Yuseong‐gu Daejeon 34141 Republic of Korea

**Keywords:** ammonia synthesis, dual‐atom catalysts, electrochemical catalysts, heterogeneous catalysts, tandem electroreduction

## Abstract

Electrochemical conversion of waste nitrate (NO_3_^−^) to ammonia (NH_3_) for environmental applications, such as carbon‐neutral energy sources and hydrogen carriers, is a promising alternative to the energy‐intensive Haber–Bosch process. However, increasing the energy efficiency is limited by the high overpotential and selectivity. Herein, a Co─Cu mixed single‐atom/cluster catalyst (Co─Cu SCC) is demonstrated—with well‐dispersed Co and Cu active sites anchored on a carbon support—that delivers high NH_3_ Faradaic efficiency of 91.2% at low potential (–0.3 V vs. RHE) due to synergism between the heterogenous active sites. Electrochemical analyses reveal that Cu in Co─Cu SCC preferentially catalyzes the NO_3_^−^‐to‐NO_2_^−^ pathway, whereupon Co catalyzes the NO_2_^−^‐to‐NH_3_ pathway. This tandem electroreduction bypasses the rate‐determining steps (RDSs) for Co and Cu to lower the reaction energy barrier and surpass scaling relationship limitations. The electrocatalytic performance is amplified by the subnanoscale catalyst to increase the partial current density of NH_3_ by 2.3 and 5.4 times compared to those of individual Co, Cu single‐atom/cluster catalysts (Co SCC, Cu SCC), respectively. This Co─Cu SCC is operated stably for 32 h in a long‐term bipolar membrane (BPM)‐based membrane electrode assembly (MEA) system for high‐concentration NH_3_ synthesis to produce over 1 m NH_3_ for conversion into high‐purity NH_4_Cl at 2.1 g day^−1^.

## Graphical abstract

A Co─Cu mixed single‐atom/cluster catalyst with well‐dispersed heterogeneous active sites is presented. These sites induce tandem nitrate electroreduction, in which Cu converts NO_3_^–^ to NO_2_^–^, after which Co catalyzes NO_2_^–^ to NH_3_ to bypass the RDS of each atom. Integration of the mixed catalyst into a long‐term BPM‐based MEA system enables high‐concentration ammonia synthesis.

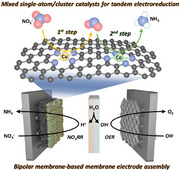

## 1 Introduction

Ammonia, a chemical compound fundamental to human civilization, has recently received attention in the environmental field because of its potential as a carbon‐neutral energy source, given its high energy density (4.32 kW h L^−1^), and its viability as an H_2_ carrier owing to its high hydrogen storage capacity (17.75%).^[^ ^1^, ^2^ ^]^ Ammonia is currently produced using the highly energy‐intensive Haber–Bosch process, which requires high temperatures (≈500 °C) and high pressures (>100 atm). This process consumes 1%–2% of global energy and produces 1.44% of CO_2_ emissions.^[^ ^3^, ^4^, ^5^ ^]^ Therefore, the development of sustainable ammonia production methods that are eco‐friendly alternatives to the Haber–Bosch process is crucial. Notably, electrochemical ammonia synthesis shows remarkable promise in this regard as it can be performed under mild conditions (room temperature, atmospheric pressure) with catalysts, resulting in less energy consumption.^[^ ^6^ ^]^ Moreover, electrolyte‐based electrochemical reactions can potentially contribute to decarbonization as they utilize water as a hydrogen source, unlike the Haber–Bosch process that relies on natural gas.^[^ ^7^, ^8^ ^]^ In particular, the electrochemical nitrate reduction reaction (NO_3_RR) is a thermodynamically active process owing to the relatively low dissociation energy of the N═O bond (204 kJ mol^−1^) compared to the N≡N bond (941 kJ mol^−1^) and has high aqueous solubility, thereby underlining its industrial feasibility.^[^ ^9^, ^10^ ^]^ Moreover, the NO_3_RR induces a purification effect as it can utilize nitrates from wastewater as a nitrogen source, thus removing harmful pollutants and enabling sustainable ammonia production by improving the management of the global nitrogen cycle.^[^ ^11^, ^12^ ^]^

(1) $${\mathrm{NO}}_{3}^{-}+\;6H_{2}O+\;8e^{-}\;\to \;NH_{3}+\;9OH^{-}$$

However, the NO_3_RR is kinetically sluggish as it typically involves the transfer of eight electrons (see the half‐cell reaction shown above).^[^ ^13^ ^]^ Additionally, the occurrence of the competitive hydrogen evolution reaction (HER) and the generation of various byproducts (NO_2_^−^, NO, NH_2_OH, N_2_O, N_2_H_2_, and N_2_) during nitrate conversion limit the efficiency and selectivity of the NO_3_RR.^[^ ^14^, ^15^ ^]^ Therefore, active catalysts that can overcome the slow NO_3_RR kinetics must be developed as they can initiate the reaction at low overpotentials and effectively suppress byproduct formation and competitive reactions, thus achieving high selectivity. Single‐atom catalysts (SACs) have emerged as promising systems in this regard owing to their maximum atomic utilization and unique electronic structures, which result in superior activity and efficient performance.^[^ ^16^, ^17^, ^18^, ^19^ ^]^ Furthermore, they exhibit high stability with strong interactions between the central atoms and the surrounding coordination atoms.^[^ ^20^ ^]^ Additionally, their distinctive configuration featuring isolated atomic active sites inhibits N–N coupling, which drives the formation of byproducts such as N_2_ and N_2_O, resulting in high selectivity toward ammonia, which makes them suitable for the NO_3_RR.^[^ ^21^, ^22^ ^]^

Recently, Cu‐based and Co‐based electrocatalysts have been comprehensively investigated for the NO_3_RR. For instance, Chen et al. designed molecular solid catalysts that incorporate various metal species, and demonstrated that Cu‐ and Co‐based catalysts exhibited the highest Faradaic efficiency (FE) in NO_3_RR‐based ammonia production.^[^ ^23^ ^]^ Cu‐based catalysts, which are known for their exceptional HER‐inhibiting features, have been widely used for the NO_3_RR owing to their superior ability to bind NO_3_^−^, thereby facilitating the catalytic conversion of NO_3_^−^ to NO_2_^−^.^[^ ^24^, ^25^, ^26^ ^]^ Similarly, using density functional theory, Carvalho et al. showed that a Co‐based SAC facilitated the conversion of NO_2_^−^ to NH_3_ owing to its favorable NO_2_^−^‐binding ability and nitric oxide dissociation capability, resulting in high ammonia production efficiency.^[^ ^27^ ^]^ These studies suggest that mixed catalysts with coexisting Co─Cu active sites within the SAC structure may exhibit outstanding catalytic performance. Moreover, the Cu and Co active sites may manifest synergistic effects, which could preferentially catalyze the NO_3_^−^ and NO_2_^−^ conversion reactions sequentially.^[^ ^28^ ^]^ The synergy derived from heterogeneous active sites may help break the linear scaling relationship of the complex multielectron/multistep NO_3_RR, which is the main limitation of SACs with only one type of active site.^[^ ^29^ ^]^ However, the design and synthesis of highly active bimetallic SACs for the NO_3_RR are in their infancy, and the mechanism governing the synergy between the heterogeneous active sites remains ambiguous.

To resolve these issues, the synergistic effect between well‐dispersed Co and Cu active sites was leveraged in this study to develop an N‐coordinated cobalt‐copper mixed single‐atom/cluster catalyst (Co─Cu SCC) that exhibited a high FE at a low overpotential. The Co─Cu SCC was synthesized using a straightforward pyrolysis method that involved calcining a mixture of metal precursors, a ligand, and carbon black. To elucidate the mechanism of interaction between the active sites in the Co─Cu SCC, Co SCC, and Cu SCC were similarly synthesized for comparison. The Co─Cu SCC demonstrated enhancements in normalized performance with respect to the onset potential, FE, and the production yield was higher than that of the Co SCC and Cu SCC, which confirms the synergy between the Co and Cu active sites. Electrochemical analyses revealed that the Cu active sites primarily catalyzed the NO_3_^−^‐to‐NO_2_^−^ conversion, whereas the Co active sites preferentially catalyzed the NO_2_^−^‐to‐NH_3_ transformation. These sequential tandem electroreductions within the Co─Cu SCC enabled the RDSs for each active site to be bypassed, thereby lowering the overall reaction energy barriers. Consequently, the Co─Cu SCC demonstrated high activity at low overpotentials to achieve an outstanding NH_3_ FE of 91.2% at –0.3 V (vs. RHE). To exploit this high catalytic performance in synthesizing high‐concentration ammonia, a long‐term‐operable BPM‐based MEA system was constructed. This system achieved an ammonia concentration exceeding 1 m through a stable 32 h reaction. Thereafter, NH_3_ was selectively extracted by air stripping to produce high‐purity NH_4_Cl powder at a rate of 2.1 g day^−1^, thus demonstrating its practical viability. The mixed SAC‐based approach and synergistic effect reported herein can be harnessed to devise flexible catalyst design strategies for the NO_3_RR.

## 2 Results and Discussion

**Figure 1:**
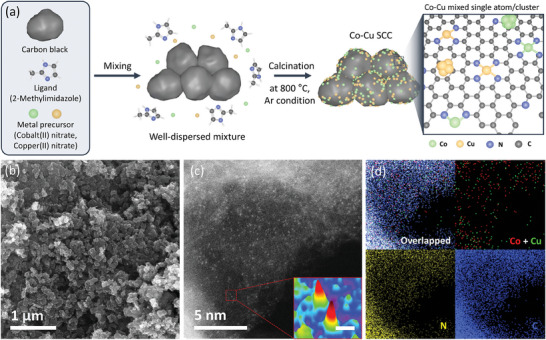
Synthesis and characterization of the cobalt‐copper mixed single‐atom/cluster catalyst (Co─Cu SCC). a) Schematic illustrating the synthesis and design of the Co─Cu SCC. b) SEM image of the Co─Cu SCC. c) High‐resolution AC‐HAADF‐STEM image of the Co─Cu SCC, indicating the formation of well‐dispersed Co and Cu atomic sites on a carbon layer surrounding carbon black, with a 3D color map of an atom pair (inset, 0.2 nm scale bar). d) EDS‐derived maps of Co, Cu, N, and C in the Co─Cu SCC.

The Co─Cu SCC, consisting of N‐coordinated atomic Co and Cu active sites with nanoclusters anchored onto carbon support, was fabricated using a ligand‐calcination‐based pyrolysis method (**Figure** **1**a). To synthesize the Co─Cu SCC, a well‐dispersed mixture comprising carbon black (Vulcan‐XC 72R) as the support, 2‐methylimidazole as the ligand for N coordination, and metal precursors were initially prepared.^[^ ^30^ ^]^ For ligand calcination, the obtained mixture was heat‐treated at 800 °C in a tube furnace under an argon atmosphere. As the ligand was calcined, N‐defective carbon layers were formed on the carbon black, and metal atoms from the precursors were anchored to the nitrogen, leading to robust metal–N–C coordination. Subsequently, this product was subjected to acid treatment to remove the metal clusters that had formed through atomic aggregation during heating. The Co─Cu SCC was fabricated using both the Co and Cu metal precursors, whereas the Co SCC and Cu SCC were synthesized using the corresponding individual metal precursor. Scanning electron microscopy (SEM) images of the Co─Cu SCC, Co SCC, and Cu SCC showed ≈50‐nm‐sized densely packed spherical structures (Figure 1b; Figure S1, Supporting Information). The Brunauer–Emmett–Teller (BET) analysis results indicated that the surface area of the Co─Cu SCC (210 m^2^ g^−1^) was comparable to that of carbon black (Table S1, Supporting Information).

High‐resolution aberration‐corrected high‐angle annular dark‐field scanning transmission electron microscopy (AC‐HAADF‐STEM) imaging revealed Co and Cu atoms anchored onto the deposited carbon layer surrounding the carbon black, confirming the formation of the atomic metal active sites using the simple pyrolysis method (Figure 1c). Identical results were obtained for the Co SCC and Cu SCC (the dispersed bright dots correspond to single metal atoms; Figure S2, Supporting Information). In the Co─Cu SCC, the metal atom sites were located at adjacent positions due to the homogeneous distribution of SAC sites, while diatomic pair configurations were also observed (highlighted in Figure S3, Supporting Information). In Figure S3 (Supporting Information), the diatomic pairs are highlighted with white boxes to clearly indicate the adjacent positions of each pair of atoms. To quantitatively confirm the distance between these pairs of atoms, we analyzed the atomic intensity profile using a line‐scanning technique. The results of this analysis reveal that the distance between the adjacent atoms is ≈0.249 ± 0.010 nm, suggesting the potential formation of atomic pairs through metal–metal bonding. However, it is important to note that distinguishing between Co and Cu atoms remains challenging because of the similarity between their atomic numbers, which result in similar Z‐contrast and atomic diameters. This similarity complicates the differentiation between the two elements when using AC‐HAADF‐STEM analysis. The inter‐site distance (*d*_site_) between each atomic active site in our Co─Cu SCC was measured using AC‐HAADF‐STEM imaging. To increase the reliability of these measurements, data points from more than 300 atoms collected by AC‐HAADF‐STEM imaging were considered for the Co─Cu SCC, resulting in an average *d*_site_ of ≈4.2 Å (Figure S4, Supporting Information). Energy‐dispersive X‐ray spectroscopy (EDS)‐based elemental mapping corroborated the uniform distribution of isolated Co and Cu active sites on the N‐doped carbon black (Figure 1d; Figure S5, Supporting Information). The amounts of Co and Cu in the synthesized Co─Cu SCC were measured by inductively coupled plasma‐mass spectrometry (ICP‐MS) to be 3.57 and 0.34 wt.%, respectively (Table S2, Supporting Information).

**Figure 2:**
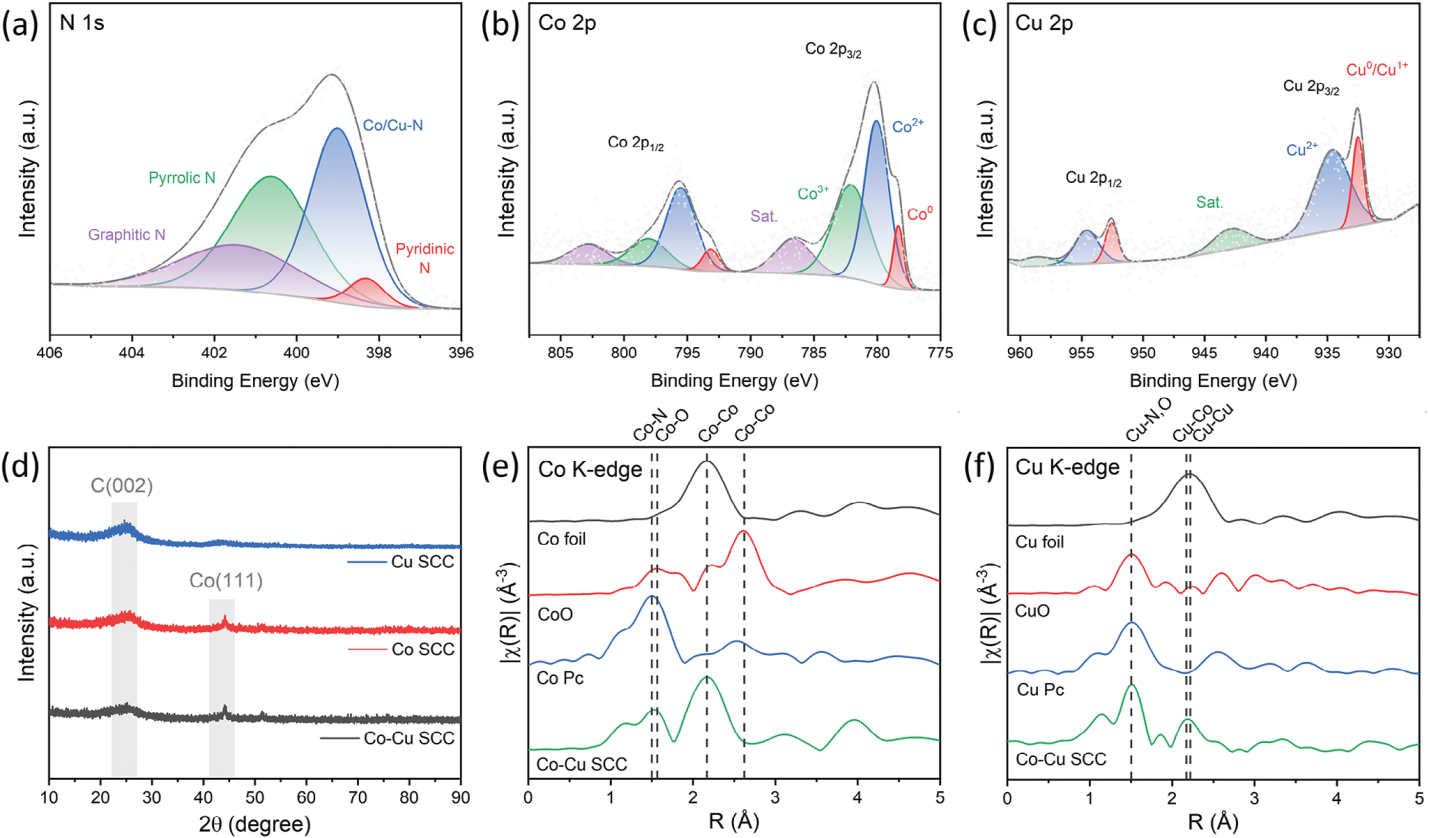
Surface and structural analyses of the Co─Cu SCC, Co SCC, and Cu SCC. a) N 1s, b) Co 2p, and c) Cu 2p XPS profiles of the Co─Cu SCC. d) High‐resolution powder XRD patterns of the Co─Cu SCC, Co SCC, and Cu SCC. e) *k* ^2^‐weighted Co K‐edge FT‐EXAFS spectra of the Co─Cu SCC, cobalt phthalocyanine (Co Pc), CoO, and Co foil. f) *k* ^2^‐weighted Cu K‐edge FT‐EXAFS spectra of the Co─Cu SCC, copper phthalocyanine (Cu Pc), CuO, and Cu foil.

X‐ray analyses were conducted to ascertain the specific single‐atom coordination mode and structures of the synthesized Co─Cu SCC (**Figure** **2**). The N 1s, Co 2p, and Cu 2p X‐ray photoelectron spectroscopy (XPS) profiles of the Co─Cu SCC were acquired (Figure 2a–c, respectively). The broad N 1s spectrum (Figure 2a) was deconvoluted into sub‐peaks, including those of pyridinic N (≈398.3 eV), pyrrolic N (≈400.6 eV), graphitic N (≈401.4 eV), and the metal–N (≈399.0 eV).^[^ ^31^, ^32^ ^]^ The N 1s subpeak at 399.0 eV, which exhibited a particularly high fraction, corresponded to Co─N or Cu─N bonds.^[^ ^33^, ^34^, ^35^ ^]^ This suggested that the nitrogen in the Co─Cu SCC was predominantly linked to metal atoms in the Co─N─C and Cu─N–C coordination structure. The O 1s spectrum from the XPS results of Co─Cu SCC showed peaks at O═C (532.6 eV) and O═C–O (533.6 eV), indicating the absence of Co─O and Cu─O coordination in the Co─Cu SCC catalyst (Figure S6, Supporting Information). In combination, the deconvolution results of the XPS N 1s and O 1s spectra and FT‐EXAFS results of Co─Cu SCC seem to suggest that Co─N and Cu─N coordination exist in the Co─Cu SCC catalyst. The Co 2p_3/2_ signals were resolved into three sub‐peaks: Co^0^ (778.3 eV), Co^2+^ (780.0 eV), and Co^3+^ (782.0 eV) (Figure 2b).^[^ ^36^ ^]^ The dominance of the 780.0 eV sub‐peak, corresponding to Co^2+^ and known to be associated with Co─N bonds, substantiated the formation of the Co─N–C coordination structure in the Co─Cu SCC.^[^ ^36^, ^37^ ^]^ Similarly, the Cu 2p_3/2_ signals were deconvoluted into Cu^0^/Cu^1+^ (932.5 eV) and Cu^2+^ (934.7 and 942.9 eV), indicating the presence of both Cu^1+^ and Cu^2+^ states, which corresponded to the Cu─N–C coordination structure (Figure 2c).^[^ ^38^, ^39^, ^40^ ^]^ The Cu^0^/Cu^1+^ peak was distinguished by Cu LMM Auger electron spectroscopy (AES), which confirmed the absence of the Co^0^ peak at 918.4 eV and the dominance of the Cu^1+^ peak at 916.6 eV on the sample surface (Figure S7, Supporting Information).^[^ ^41^ ^]^ To improve the resolution of the Cu 2p XPS spectrum and Cu LMM Auger electron spectrum of the Co─Cu SCC, ion beam etching using Ar sputtering was used to remove surface contaminants, such as adsorbed organic molecules, which could interfere with the XPS analysis and potentially obscure the Cu signal (Figure S8, Supporting Information). However, despite this additional etching process, the signal intensities for Cu 2p XPS spectrum and Cu LMM Auger electron spectrum remained low. This was ascribed to the low Cu content of 0.1 at% in Co─Cu SCC (Table S3, Supporting Information) being near the detection limit.^[^ ^42^ ^]^ The XPS analyses of the Cu SCC and Co SCC provided similar results, and validated the presence of Co─N and Cu─N bonds (Figures S9 and S10, Supporting Information).

The powder X‐ray diffractometry (XRD) patterns of all the synthesized catalysts (Figure 2d) had a broad peak at ≈24°, which corresponds to the (002) planes of graphite.^[^ ^20^, ^21^ ^]^ Although exposure to the acid removed the exposed metal clusters, faint metallic peaks, such as those of Co (111) at 44.2° and Co (200) at 51.5°, indicated that embedded clusters were still present. The persistence of metal clusters was due to the formation of carbon shells surrounding nanoclusters during heat treatment (Figure S11, Supporting Information). These carbon shells hindered the penetration of the etching solution and prevented the complete elimination of the metal clusters (Figure S12, Supporting Information).^[^ ^43^ ^]^ Previous studies have shown that residual metal clusters were inactivated by the encapsulating carbon shells.^[^ ^43^, ^44^, ^45^, ^46^ ^]^ To verify that the carbon‐coated clusters in Co─Cu SCC were not involved in the reactions, a controlled experiment was conducted to compare the NO_3_RR performance based on the extent of cluster inclusion, which was controlled by varying the acid treatment time. The sample for comparison was synthesized under the same conditions to maintain the same amount of SAC and Co/Cu ratio on the surface, but the acid treatment time was shortened to increase the metal cluster content. This increased the metal content by 28% according to inductively coupled plasma optical emission spectroscopy (ICP‐OES) and intensified the metal peaks on the XRD pattern (Figure S13, Supporting Information). Despite the difference in cluster content, the NO_3_RR performances were identical (Figure S14, Supporting Information). This result indicates that in Co─Cu SCC, the reaction predominantly occurred at the SAC sites on the surface, and the metal clusters encapsulated with a carbon shell were involved to a limited extent.

Fourier‐transformed extended X‐ray absorption fine structure (FT‐EXAFS) analysis was conducted to precisely confirm the existence of metal active sites with the SAC structure. *k* ^2^‐weighted Co and Cu K‐edge EXAFS data were acquired in R space for the Co─Cu SCC with reference samples (Figure 2e,f). The Co K‐edge showed a distinct peak at 1.5 Å, corresponding to the first shell scattering of the Co─N coordination structure; this is consistent with the state of Co atoms in cobalt phthalocyanine (Co Pc) with Co─N_4_ structures (Figure 2e).^[^ ^47^, ^48^, ^49^ ^]^ Another intense peak, observed at 2.15 Å, was attributed to the Co─Co bond and/or Co─Cu bond, which could be induced by the presence of residual metal clusters not involved in the reaction. The Cu K‐edge showed a dominant peak at 1.48 Å, which corresponded to the Cu─N coordination structure, indicating that the Cu atoms in the Co─Cu SCC had local structures equivalent to those in copper phthalocyanine (Cu Pc), which exhibited the Cu─N_4_ configuration (Figure 2f).^[^ ^22^, ^50^ ^]^ The second peak of the Co─Cu SCC at 2.17 Å, which was absent from the Cu SCC result, is shifted to a lower R than the first shell distance of the Cu─Cu bond (2.20 Å) and is higher than that of the distance of the Co─Co bond (2.15 Å). This suggests that it represents the Cu─Co bond and potentially involves a diatomic configuration as depicted in the AC‐HAADF‐STEM image (Figure 1c) or Co clusters alloyed with Cu.^[^ ^31^, ^51^ ^]^ The Co─N, Cu─N, Co─Co, and Cu─Cu bonds were also observed for the Co SCC and Cu SCC upon FT‐EXAFS analysis (Figure S15, Supporting Information). This finding, which indicated the presence of Co/Cu─N configurations with residual nanoclusters, validated the successful synthesis of the Co─Cu mixed SAC structures using ligand‐calcination‐based pyrolysis.

**Figure 3:**
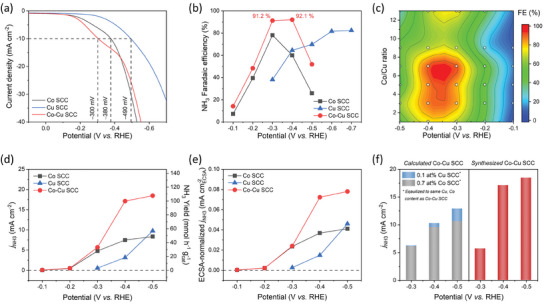
Optimization of reaction conditions and performance of the electrocatalytic nitrate reduction reaction (NO_3_RR). a) LSV curves and b) potential‐dependent NH_3_ Faradaic efficiency (FE) of the Co─Cu SCC, Co SCC, and Cu SCC in an electrolyte containing 0.1 m KOH and 0.1 m KNO_3_. c) Contour plot of the NH_3_ FE for the Co─Cu SCC with various Co/Cu molar ratios as a function of potential. d) NH_3_ partial current density and NH_3_ yield for each catalyst as a function of potential, along with normalized results based on e) ECSA and f) surface metal atomic content obtained by XPS analysis.

The interactions between the Co and Cu active sites were investigated by comparing the NO_3_RR performances of the Co─Cu SCC with those of the Co SCC and Cu SCC (**Figure** **3**). To that end, electrochemical NO_3_RR tests were performed in a customized H‐type cell with a Hg/HgO reference electrode under alkaline conditions (pH 13) with 0.1 m KOH and 0.1 m KNO_3_. The synthesized‐catalyst‐loaded carbon paper and platinum foil were employed as the working and counter electrodes, respectively. The concentrations of compounds produced via the NO_3_RR, such as NH_3_, NO_2,_ and NH_2_OH, were quantitatively determined using a colorimetric method based on ultraviolet‐visible (UV‐vis) spectrophotometry (Figure S16, Supporting Information). This colorimetric method was validated through a quantification technique based on ^1^H nuclear magnetic resonance (NMR) analysis, which involved calibrating the normalized NH_4_^+^ peak integration relative to the internal standard (Figure S17, Supporting Information).^[^ ^31^, ^52^ ^]^ The NH_3_ yield results obtained from the two quantification methods showed substantial agreement, validating the reliability of the UV‐vis spectrophotometry‐based colorimetric measurement scheme established to evaluate the ammonia production efficiency. Additionally, ^1^H‐NMR‐based isotope labeling analysis (Figure S17a, Supporting Information) confirmed that the ammonia was derived from the NO_3_^−^ reactant and not from any contamination or catalyst interference.^[^ ^24^ ^]^ The absence of peaks corresponding to ammonia from the ^1^H‐NMR spectrum of the pre‐reaction electrolyte excluded the influence of contamination. In addition, reaction with the isotope ^15^NO_3_^−^ reactant showed the absence of a triplet signal corresponding to ^14^NH_4_^+^ and only exhibited a doublet signal corresponding to ^15^NH_4_^+^, and verified that ammonia was solely generated through the electrochemical conversion of nitrate.^[^ ^53^, ^54^ ^]^

Linear sweep voltammetry (LSV) curves were recorded to determine the onset potential and NO_3_RR catalytic activity for the Co─Cu SCC, Co SCC, and Cu SCC (Figure 3a). The Co─Cu SCC demonstrated a notably lower overpotential for initiating the reaction than those of the Co SCC and Cu SCC, leading to a higher current density within the potential range where the NO_3_RR predominantly occurred (–0.1 to –0.5 V (vs. RHE)). In particular, the Co─Cu SCC achieved a current density of 10 mA cm^−2^ at –300 mV (vs. RHE), which was 80 and 190 mV earlier than when the Co SCC and Cu SCC achieved it, respectively. This indicated that the lower energy barrier of the NO_3_^−^‐to‐NH_3_ conversion resulting from the interactions between the Co and Cu active sites enhanced the onset potential of the Co─Cu SCC compared with that of the monometallic SCCs.

The NH_3_ FE was investigated against the potential by conducting 30 min NO_3_RR tests with the Co─Cu SCC, Co SCC, and Cu SCC (Figure 3b). The FE of the Co SCC exhibited a peak with a maximum value of 78.1% at –0.3 V (vs. RHE), indicating reaction initiation at a lower overpotential than that of the Cu SCC, as is evident from the LSV results; however, it tended to be susceptible to the HER at higher potentials. Conversely, the Cu SCC retained a high efficiency of 82.4% at –0.7 V (vs. RHE) owing to its inertness to the HER, but it required high potential to initiate the NO_3_RR. The Co─Cu SCC, which showed hybrid characteristics derived from both monometallic SCCs, exhibited superior performance at low potentials as well as significant HER suppression, thus exhibiting a remarkable NH_3_ FE of 91.2% at a low potential of –0.3 V (vs. RHE). Furthermore, at –0.4 V (vs. RHE), the NH_3_ FE of the Co─Cu SCC was 1.5‐ and 1.4‐fold higher than those of the Co SCC and Cu SCC, respectively.

A Co/Cu molar ratio of 7 led to optimal Co─Cu SCC performance with respect to the NH_3_ FE, as indicated by the FE contour plot obtained as a function of the Co/Cu ratio and potential (Figure 3c). Consequently, all Co─Cu SCC specimens were fabricated with a Co/Cu ratio of 7, as confirmed by the XPS‐based surface content analysis (Table S3, Supporting Information), which showed that the synthesized samples comprised 0.7 at% Co and 0.1 at% Cu. The NH_3_ partial current density, which was proportional to the NH_3_ yield, clearly demonstrated the enhanced activity and selectivity of the Co─Cu SCC over those of the Co SCC and Cu SCC (Figure 3d). In particular, the Co─Cu SCC exhibited a higher NH_3_ partial current density than those of the monometallic SCCs at all analyzed potentials, with values that were 2.3‐ and 5.4‐fold higher than those of the Co SCC and Cu SCC, respectively, at –0.4 V (vs. RHE). Notably, if the reaction had occurred individually at the Co and Cu active sites in the Co─Cu SCC, the performance would have converged to the average values of the Co SCC and Cu SCC. Therefore, the improved NO_3_RR of the Co─Cu SCC implied the presence of a synergistic effect between the Co and Cu active sites.

Normalized NH_3_ partial current densities were obtained to determine whether the enhanced activity and selectivity of the Co─Cu SCC were due to extrinsic factors such as the surface area and metal content (Figure 3e,f). The electrochemical surface area (ECSA) of each catalyst was used to normalize the NH_3_ partial current density to the effective surface area. The ECSAs of the synthesized catalysts were determined by measuring the electrochemical double‐layer capacitance (C_dl_) values through cyclic voltammetry (CV) performed in non‐Faradaic potential windows (Figure S18, Supporting Information). The Co─Cu SCC, Co SCC, and Cu SCC exhibited similar ECSAs (236.8, 203.2, and 212.2 cm^2^, respectively), with the Co─Cu SCC showing a higher ECSA‐normalized NH_3_ partial current density than those of the monometallic SCCs in the –0.4 to –0.5 V region (vs. RHE; Figure 3e). This indicated that the performance enhancement of the Co─Cu SCC was independent of the catalyst surface area. Similarly, the XPS‐analysis‐derived atomic percentages of metallic elements on the surface were used to normalize the NH_3_ partial current density with respect to the metal contents (Table S3, Supporting Information). To achieve metal content equivalence with the Co─Cu SCC, the NH_3_ partial current densities of the Co SCC and Cu SCC were normalized to 0.7 at% Co and 0.1 at% Cu, respectively. Subsequently, the normalized Co SCC and Cu SCC results were summed to obtain the NH_3_ partial current density of a hypothetical calculated sample with the same metal contents as those in the Co─Cu SCC but with distinctly separated Co and Cu active sites (Figure 3f). Despite having the same surface metal contents, the Co and Cu active sites exhibited a significantly higher NH_3_ partial current density under the mixed condition (synthesized Co─Cu SCC) than in the separated case (calculated Co─Cu SCC) in the range –0.4 to –0.5 V (vs. RHE). This indicated that the performance enhancement of the Co─Cu SCC was not solely due to extrinsic properties such as the surface area and metal content, but rather to the synergy between the Co and Cu active sites, which improved its intrinsic capabilities.

**Figure 4:**
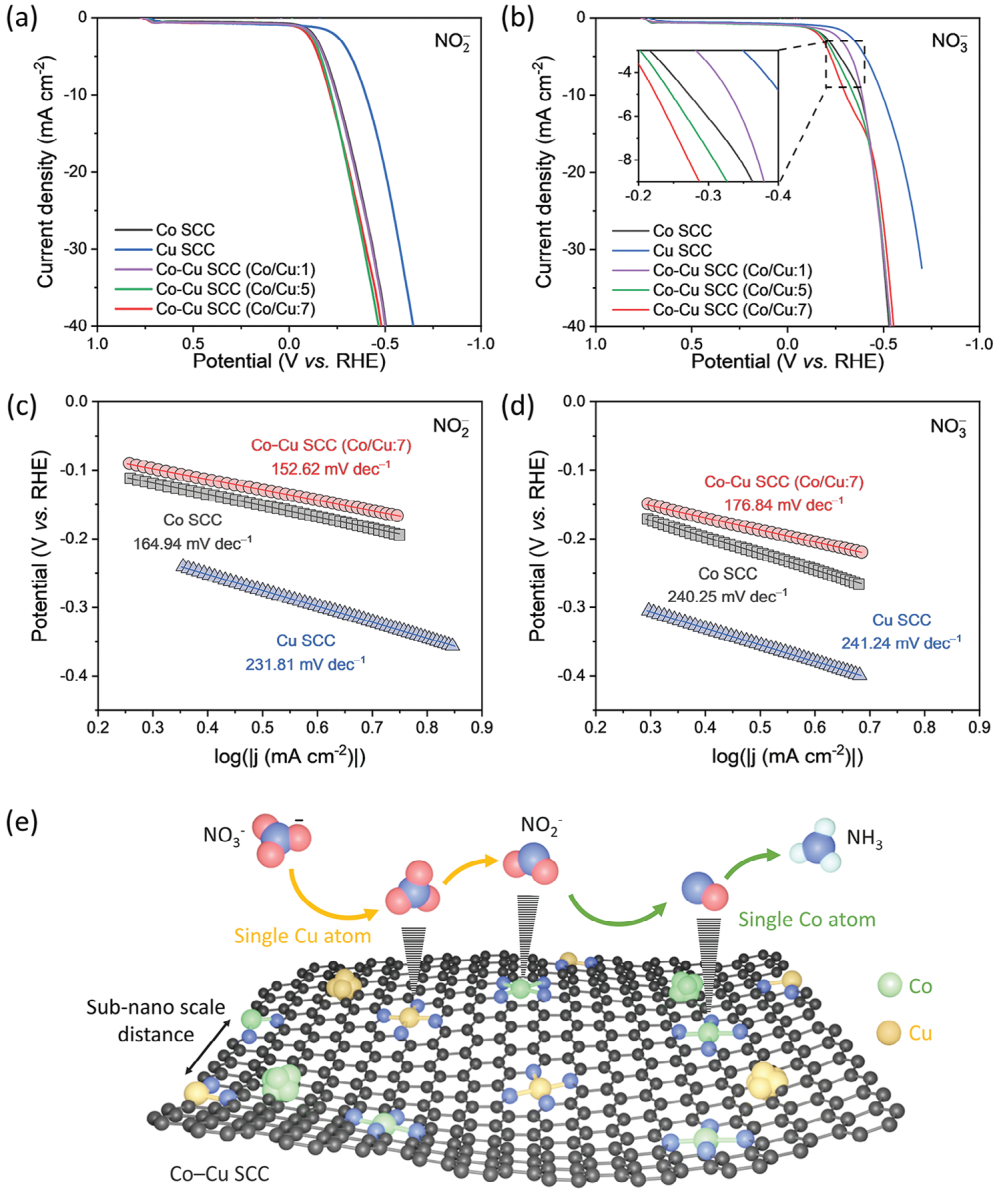
Reaction kinetics and synergistic mechanism for the Co─Cu SCC. LSV curves were acquired using a) 0.1 m KNO_2_ and b) 0.1 m KNO_3_ for the Co SCC, Cu SCC, and Co─Cu SCC with several Co/Cu ratios. LSV‐derived Tafel plots were constructed using c) 0.1 m KNO_2_ and d) 0.1 M KNO_3_ for the Co─Cu SCC, Co SCC, and Cu SCC. e) Schematic depicting tandem electroreduction of nitrate to ammonia using the Co─Cu SCC.

To elucidate the synergistic mechanism underlying the interactions between the Co and Cu active sites in the Co─Cu SCC, additional electrochemical analyses were performed using NO_2_^−^ and NO_3_^−^ as reactants (**Figure** **4**). LSV curves of the Co SCC, Cu SCC, and Co─Cu SCC with various Co/Cu ratios (1, 5, and 7) were acquired using 0.1 m NO_2_^−^ and 0.1 m NO_3_^−^ as reactants (Figure 4a,b, respectively). When NO_2_^−^ was the reactant (Figure 4a), the LSV results for the Co SCC and Co─Cu SCCs were almost identical regardless of the presence of Cu active sites, indicating that the NO_2_^−^‐to‐NH_3_ conversion predominantly occurred at the Co active sites rather than the Cu active sites. Conversely, the LSV results obtained for the Co SCC and Co─Cu SCCs using NO_3_^−^ as the reactant (Figure 4b) differed in terms of the onset potential and current density owing to the inclusion of Cu active sites in the NO_3_RR potential region. Notably, the presence of Cu active sites induced differences in the LSV curves only for the NO_3_^−^ reactant and not for NO_2_^−^. This suggested that in the entire NO_3_^−^ → NO_2_^−^ → NH_3_ pathway, the NO_3_^−^‐to‐NO_2_^−^ conversion preferentially occurred at the Cu active sites. Therefore, in the Co─Cu SCC, the NO_3_^−^‐to‐NO_2_^−^ reduction was catalyzed at the Cu active sites, followed by the NO_2_^−^‐to‐NH_3_ reduction that was primarily catalyzed at the Co active sites.

Electrokinetic analyses were performed by constructing Tafel plots using NO_2_^−^ and NO_3_^−^ as reactants to determine the RDS, thus clarifying the relationship between the sequential favorable reactions proceeding at the heterogeneous active sites and the improvements in mixed SCC performance. The Tafel plots corresponding to 0.1 m NO_2_^−^ (Figure 4c) and 0.1 m NO_3_^−^ (Figure 4d) showed that the Tafel slope of the Co─Cu SCC was smaller than those of the Co SCC and Cu SCC, indicating that the mixed SCC was more active than the monometallic SCCs. Additionally, the Tafel slope of the Co SCC was significantly lower for the NO_2_^−^‐to‐NH_3_ conversion (164.94 mV dec^−1^) than that for the NO_3_^−^‐to‐NH_3_ transformation (240.25 mV dec^−1^). The higher activity for the NO_2_^−^‐to‐NH_3_ conversion than that for the entire NO_3_^−^‐to‐NH_3_ process indicated that the sluggish NO_3_^−^‐to‐NO_2_^−^ reaction limited the overall process. This demonstrated that the RDS for the Co active sites was located along the NO_3_^−^‐to‐NO_2_^−^ conversion pathway. Furthermore, the Tafel slope of the Cu SCC had similar values for the NO_3_^−^‐to‐NH_3_ and NO_2_^−^‐to‐NH_3_ reactions (241.24 and 231.81 mV dec^−1^, respectively), suggesting that the RDS for Cu active sites was located along the NO_2_^−^‐to‐NH_3_ conversion pathway, which was present in both reactions. This N‐coordinated Cu SAC active site was known to aggregate into Cu clusters under potential‐driven conditions, suggesting that the reaction kinetics could be influenced by the restructured surface.^[^ ^55^ ^]^ The synergistic effect between the heterogeneous active sites in the Co─Cu SCC allowed the RDSs of Co active sites (in the NO_3_^−^‐to‐NO_2_^−^ conversion) and those of Cu active sites (in the NO_2_^−^‐to‐NH_3_ conversion) to be circumvented by the sequential tandem NO_3_RR in which Cu active sites converted NO_3_^−^ to NO_2_^−^ and Co active sites transformed NO_2_^−^ to NH_3_ (Figure 4e). Consequently, the energy barrier of the overall reaction was lowered by the complementary reactions at the Co and Cu active sites in the Co─Cu SCC to improve the catalytic NO_3_RR performance.

This mechanism was boosted by the shortened distance between the heterogeneous active sites, which facilitated the transfer of intermediate NO_2_^−^. This was verified by comparing the post‐synthesis mixing sample (combination of Co SCC and Cu SCC) and the pre‐synthesis mixing sample (Co─Cu SCC), where it was demonstrated that shorter distances between heterogeneous active sites increased the synergy, which increased the NH_3_ partial current density (Figure S19, Supporting Information). A subsequent investigation of whether the synergy of Co─Cu SCC is based on NO_2_^−^ spillover‐driven tandem reduction leveraged the principle that the spillover mechanism is governed by the distance over which intermediates are transferred and that this effect diminishes as the distance between active sites increases.^[^ ^26^, ^56^ ^]^ By controlling the amount of metal precursor in Co─Cu SCC, the distance between atomic sites was successfully adjusted, which increased the spacing, as is clear from the AC‐HAADF‐STEM images (Figure S20, Supporting Information). The metal content on each electrode was standardized to 0.03 mg cm^−2^ by regulating the loading mass, thereby ensuring that performance variations were solely attributable to the inter‐distance between active sites rather than to the quantity of active sites. As the inter‐site distance increased, the electrocatalytic performance, including the mass activity, NH_3_ FE, and NH_3_ partial current density decreased, and this indicated the involvement of spillover in tandem catalysis (Figure S21, Supporting Information).^[^ ^56^, ^57^ ^]^ Similarly, the increase in NO_2_^−^ selectivity relative to NH_3_ as the inter‐site distance increased could be attributed to the suppression of the spillover effect by the greater distance, which inhibited subsequent reactions, resulting in the desorption of NO_2_^−^ from the catalyst surface. Consequently, the synergistic effect of the Co─Cu SCC was enhanced due to the close proximity of active sites, particularly with the SAC structure existing at subnanometer spacing.

**Figure 5:**
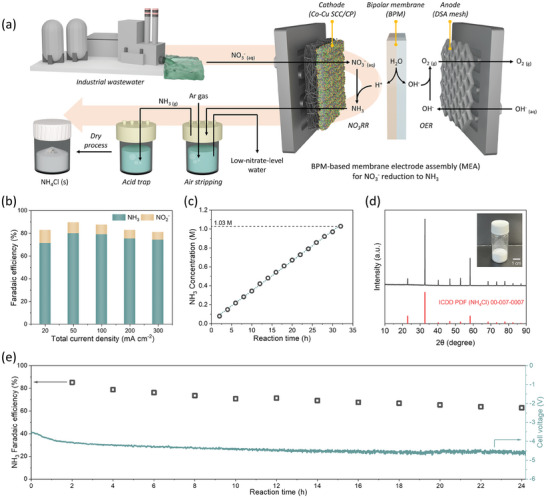
Application of the Co─Cu SCC in a membrane electrode assembly (MEA) system. a) Schematics of the bipolar‐membrane (BPM)‐based MEA system and the underlying nitrogen conversion scheme. b) NH_3_ and NO_2_^–^ FEs of the Co─Cu SCC over a wide range of current densities in the MEA system with 2 m KNO_3_ and 1 m KOH. Results of long‐term MEA operation conducted at 100 mA cm^–2^; c) concentration of produced NH_3_ as a function of reaction time, d) XRD pattern and digital photograph of synthesized NH_4_Cl salt, and e) reaction stability with respect to NH_3_ FE and cell voltage for a 24 h reaction.

To scale up the production of ammonia, the Co─Cu SCC was integrated into a 5 cm^2^ zero‐gap‐based MEA system with a catalyst‐coated substrate (CCS) structure. In contrast to H‐type cells, MEA systems can achieve higher current densities owing to their lower internal resistance and inclusion of reactant cycling systems. In addition, they are readily scalable for larger applications, which makes them suitable for verifying the feasibility for empirical utilization.^[^ ^58^ ^]^ An MEA system for larger scale NH_3_ production was constructed by loading the Co─Cu SCC onto carbon paper (5 cm^2^) as the cathode, using commercial dimensionally stable anode (DSA) mesh as the anode, and a BPM (**Figure** **5**a). The BPM, which comprised a cation‐exchange layer (CEL) and an anion‐exchange layer (AEL), induced water dissociation at the layer interface when voltage was applied, thus generating H^+^/OH^−^ pairs. In this setup, the CEL is placed on the cathode side and utilizes the H^+^ generated from the BPM for the hydrogenation of NO_3_^−^ to NH_3_. Simultaneously, OH^−^ was generated via the BPM flow to the anode and then utilized in the oxygen evolution reaction. This process facilitates protonation in the NO_3_RR and lowers the reaction energy barrier but also induces the HER, a well‐known competing reaction.^[^ ^59^ ^]^ Reportedly, an excess of H^+^ generated from the BPM can lead to HER, underscoring the importance of balancing the consumption of H^+^ by NO_3_RR with the H^+^ supply from the water dissociation reaction in the BPM.^[^ ^59^ ^]^ To confirm the effect of the H^+^ supply, we conducted NO_3_RR tests with the Co─Cu SCC with different electrolytes. As depicted in Figure S22 (Supporting Information), NO_3_RR tests were performed in 0.1 m KOH + 1 m KNO_3_ and 1 m KOH + 1 m KNO_3_ as the electrolytes. The results showed that when the pH increased from 0.1 m KOH to 1 m KOH, the NH_3_ FE of Co─Cu SCC also increased due to the suppression of the excessive H^+^ supply. These findings clearly indicate that the reaction conditions need to be optimized for the zero‐gap‐based MEA system with a BPM.

The BPM‐based MEA system was used to establish a process that converted wastewater‐derived NO_3_^−^ into NH_4_Cl salt, a high‐value‐added substance produced from NH_3_ (Figure 5a). The operation was performed using 2 m KNO_3_ with 1 m KOH as the electrolyte (50 mL); this composition is similar to that of low‐level nuclear wastewater.^[^ ^60^ ^]^ Subsequently, the FEs of NH_3_ and NO_2_^−^ were determined with respect to the total current density derived from a 30 min reaction by chronopotentiometry (Figure 5b). Even at higher current densities than those of H‐type cells, an NH_3_ FE of over 70% was sustained, with a respectable value of 74.3% achieved at 300 mA cm^−2^ (1.5 A). To obtain high‐concentration NH_3_, the 5 cm^2^ MEA system was operated at 100 mA cm^−2^ for an extended period, resulting in concentrations of 0.8 m and over 1 m after 24 and 32 h, respectively (Figure 5c). Additionally, the high linearity (R^2^ = 0.9993) of the NH_3_ concentration–time plot underscored the potential for achieving even higher NH_3_ concentrations. For demonstrative purposes, an additional synthesis process was established to extract NH_3_ in the form of ammonium chloride (NH_4_Cl_(s)_) from the high‐concentration ammonia‐containing electrolyte that had reacted for 1 day (Figure 5a). To that end, inert argon gas (100 sccm) was bubbled into the NH_3_‐containing reacted electrolyte to selectively extract NH_3_ by air stripping. The volatilized NH_3(g)_ was captured in an HCl solution to form NH_4_Cl_(s)_, which was then dried in a rotary evaporator to yield 2.1 g of white high‐purity NH_4_Cl_(s)_ powder after a 24 h reaction (Figure S23, Supporting Information), as confirmed by XRD (Figure 5d). Moreover, the NH_3_ FE and cell voltage remained relatively stable during the 24 h reaction (Figure 5e; results of the 24‐h stability test), and value‐added NH_4_Cl_(s)_ powder was synthesized from wastewater at a production rate of 2.1 g day^−1^. Thus, a complete empirical system was implemented to convert nitrate to a value‐added NH_3_‐related product (NH_4_Cl_(s)_) using the Co─Cu SCC with an MEA cell, which can be utilized for wastewater purification and ammonia production purposes through electrocatalytic reactions.

## 3 Conclusion

A Co─Cu SCC comprising a carbon black support decorated with well‐dispersed Co and Cu atoms was synthesized using a facile calcination method. The bimetallic mixed SCC delivered enhanced NO_3_RR performance owing to the synergistic effect of the heterogeneous active sites. The results of our electrochemical analysis revealed that the atomic Cu active sites preferentially catalyzed the NO_3_^−^‐to‐NO_2_^−^ conversion, and the atomic Co active sites catalyzed the subsequent NO_2_^−^‐to‐NH_3_ transformation. The sequential tandem conversion reactions in the Co─Cu SCC helped bypass the RDS for each Co and Cu active site, thereby reducing the reaction energy barrier of the overall conversion pathway. The underlying mechanism was boosted by SAC‐containing structures with subnanoscale distances between the active sites. Consequently, the Co─Cu SCC exhibited high NO_3_RR performance with high selectivity (91.2% NH_3_ FE at –0.3 V vs. RHE) and advanced activity (the NH_3_ partial current density was 2.3‐ and 5.4‐fold higher than those of the Co SCC and Cu SCC, respectively, at –0.4 V vs. RHE). Finally, the Co─Cu SCC was incorporated into a long‐term‐operable BPM‐based MEA system to produce high‐concentration NH_3_ (>1 m), which was successfully converted to high‐purity NH_4_Cl powder (2.1 g day^−1^). Overall, this report presents a flexible design approach for single‐atom/cluster catalysts and demonstrates their practical application by integrating them into an MEA system, thus offering a viable synergy‐enhancing strategy based on the structural engineering of heterogeneous active sites.

## Conflict of Interest

The authors declare no conflict of interest.

## Supporting information



Supporting Information

## Data Availability

The data that support the findings of this study are available from the corresponding author upon reasonable request.
