# Supplementary material for: Tandem Electroreduction of Nitrate to Ammonia Using a Cobalt–Copper Mixed Single‐Atom/Cluster Catalyst with Synergistic Effects
Source: Adv Sci (Weinh). 2024 Sep 19;11(42):2407250. doi: 10.1002/advs.202407250 (PMC11558078; doi:10.1002/advs.202407250)
Supplement: Supplementary file 1 — Supporting Information [file ADVS-11-2407250-s001.docx]

Supporting Information

**Tandem Electroreduction of Nitrate to Ammonia Using a Cobalt-Copper Mixed Single-atom/Cluster Catalyst with Synergistic Effects**

*Jungwon Suh,^†^ Hyeonuk Choi,^†^ Yujin Kong, and Jihun Oh**

*† J. S. and H. C. contributed equally to this paper.*

*Corresponding author

E-mail: jihun.oh@kaist.ac.kr

**J. Suh, H. Choi, Y. Kong, J. Oh**

Department of Materials Science and Engineering, Korea Advanced Institute of Science and Technology (KAIST), 291 Daehak-ro, Yuseong-gu, Daejeon 34141, Republic of Korea

E-mail: jihun.oh@kaist.ac.kr

**EXPERIMENTAL SECTION**

**Materials**

Cobalt(II) nitrate hexahydrate (Co(NO_3_)_2_·6H_2_O, ACS reagent grade, ≥98%), copper(II) nitrate trihydrate (Cu(NO_3_)_2_·3H_2_O, puriss. p. a., 99%–104%), 2-methylimidazole (C_4_H_6_N_2_, 99%), potassium hydroxide (KOH, ≥85%), potassium nitrate (KNO_3_, ReagentPlus®, ≥99.0%), sodium citrate tribasic dihydrate (HOC(COONa)(CH_2_COONa)_2_·2H_2_O, ACS reagent grade, ≥99.0%), salicylic acid (2·(HO)C_6_H_4_CO_2_H, ACS reagent grade, ≥99.0%), a sodium hypochlorite (NaOCl) solution (reagent grade, 4.00%–4.99%), sulfanilamide (H_2_NC_6_H_4_SO_2_NH_2_, ≥98%), N-(1-naphthyl) ethylenediamine dihydrochloride (C_10_H_7_NHCH_2_CH_2_NH_2_·2HCl, ACS reagent grade, >98%), sodium nitroferricyanide(III) dihydrate (Na_2_[Fe(CN)_5_NO]·2H_2_O, ACE reagent, ≥99%), Nafion® 117 solution (5 wt% solution), 8-quinolinol (C_9_H_7_NO, ACS reagent grade, ≥99%), phosphoric acid (H_3_PO_4_, ASC reagent grade, ≥85 wt% in H_2_O), trichloroacetic acid (Cl_3_CCOOH, ACS reagent grade, ≥99.0%), and a nitrate ion standard solution (0.01 M NO_2_^−^ for ion-selective electrodes) were obtained from Sigma Aldrich. Hydrochloric acid (HCl, 36%) and sodium hydroxide (NaOH, 97%) were acquired from Junsei. Carbon black (Vulcan XC72R) was procured from Fuel Cell Store. Ultrapure deionized (DI) water (18.2 MΩ·cm, Millipore Milli-Q system) and ethanol (CH_3_CH_2_OH, OCI Company, 94%) were used in all the experimental procedures.

**Synthesis of catalysts**

To synthesis the Co-Cu mixed single-atom/cluster catalyst (Co-Cu SCC), Co SCC, and Cu SCC, a well-dispersed mixture of carbon black, the ligand (2-methylimidazole), and metal precursors (cobalt(II) nitrate hexahydrate and/or copper(II) nitrate trihydrate) was calcined. Subsequently, Vulcan-XC72R carbon black (200 mg) was dispersed in ethanol (60 mL) added to a round-bottom flask by ultrasonication. The metal precursors and 2-methylimidazole (135.4 mg) were respectively dissolved in ethanol (20 mL) and then added to the carbon black solution. As metal precursors, cobalt(II) nitrate hexahydrate (104.9 mg) and copper(II) nitrate trihydrate (12.4 mg) were used to produce the Co-Cu SCC, whereas cobalt(II) nitrate hexahydrate (119.9 mg) and copper(II) nitrate trihydrate (99.2 mg) were used to synthesize the Co SCC and Cu SCC, respectively. The mixed solution was sonicated for 20 min to obtain a homogeneous dispersion. The resulting mixture was placed in a rotary evaporator (250 rpm, 65 °C, 0.7 atm) for 1 h to remove the ethanol and then heated at 80 °C in a box oven for 1 h, yielding a well-dispersed black solid. The obtained material was dried, pulverized, and then heat-treated in a tube furnace at 800 °C for 2 h using a temperature ramp rate of 5 °C min^−1^ under an argon atmosphere to calcine 2-methylimidazole, leading to metal–N–C coordination. The product was acid-treated with HCl (7 M, 200 mL) under ultrasonication for 6 h to eliminate the partially generated metal nanoparticles. The resulting material was neutralized with DI water to prepare powder-form catalysts. Additionally, the post-synthesis mixing sample (Figure S18) was prepared by mixing the synthesized Co SCC and Cu SCC in a 7:1 ratio, corresponding to the Co, Cu ratio in Co-Cu SCC.

**Electrochemical measurements**

The NO_3_RR for electrochemical ammonia production was conducted in an H-type cell setup, which was separated by a Nafion membrane (N-117 proton exchange membrane). The working cell contained 0.1 M KOH and 0.1 M KNO_3_ as the electrolyte (55 mL), whereas the counter cell contained 0.1 M KOH as the electrolyte (55 mL). Platinum foil (2 × 1 cm^2^) and a Hg/HgO electrode (RE-61AP, Biologic) containing 0.1 M KOH were used as the counter and reference electrodes, respectively. The working electrode with a reaction area of 1 × 1 cm^2^ was fabricated by loading the synthesized powder catalyst (0.8 mg cm^−2^) onto carbon paper (39BB, Sigracet) by hand spraying. During the reaction, the electrolyte in the working cell was agitated with a stirrer bar spinning at 150 rpm. An SP-300 potentiostat (Biologic) was used to apply potential and current to the cell, with the applied potentials measured using the Hg/HgO reference electrode and subsequently converted to the reversible hydrogen electrode (RHE) scale using the equation

E (V *vs.* RHE) = E (V *vs.* Hg/HgO) + 0.098 + 0.059 × pH.

The electrocatalytic performance of each synthesized catalyst in the NO_3_RR was evaluated by linear sweep voltammetry (LSV) and chronoamperometry (CA). Potentiostatic electrochemical impedance spectroscopy (PEIS) with 85% iR compensation was performed prior to each electrochemical reaction to apply ohmic drop correction. LSV was conducted from 0 to −2.0 V (*vs.* RHE) at a scan rate of 20.0 mV s^−1^ to discern the current–voltage relationship. CA was performed during the NO_3_RR tests at a certain stationary potential for 30 min to evaluate electrocatalytic performance attributes such as the Faradaic efficiency (FE) and NH_3_ yield rate.

**Detection of NH_3_**

The NH_3_ concentration was determined using a previously reported indophenol blue method.^[1]^ For quantification, the reacted electrolyte (2 mL) was first added to a 5 mL tube. Subsequently, a mixture of a NaOH solution (2 mL) containing salicylic acid and sodium citrate tribasic dihydrate and a NaOCl solution (1 mL) was added to the preceding solution, and the resulting system was incubated for 20 min after being thoroughly mixed. A sodium nitroferricyanide (III) solution (200 µL) was added thereafter, and the obtained mixture was incubated for an additional 2 h under dark conditions. Subsequently, the absorbance at 655 nm—which corresponded to the formation of indophenol blue—was determined using an ultraviolet-visible (UV-Vis) spectrophotometer (OPTIZEN Alpha, KLAB). The obtained absorbance values were then quantitatively compared with the concentration–absorbance relationship established by calibration (Figure S15a) to determine the concentration of the resulting NH_3_.

**Detection of NO_2_^−^**

The NO_2_^−^ concentration was measured using a standard method based on UV-Vis spectrophotometry. To that end, the reacted electrolyte was diluted 50-fold, and the resulting solution (4 mL) was transferred to a 5 mL tube. Subsequently, a color reagent for NO_2_^−^ (40 µL)—comprising sulfanilamide, N-(1-naphthyl) ethylenediamine dihydrochloride, and phosphoric acid—was added. The mixture was incubated for 15 min, and the absorbance at 540 nm was measured thereafter by UV-Vis spectrophotometry. The obtained absorbance values were then quantitatively compared with the calibration results (Figure S15b) to determine the concentration of the NO_2_^−^ intermediate.

**Detection of NO_3_^−^**

The NO_3_^−^ concentration was determined using a standard method based on UV-Vis spectrophotometry. The electrolyte was diluted 50-fold, and the resulting solution (4 mL) was transferred to a 5 mL tube along with HCl (1 M, 200 µL) and sulfamic acid (0.8 wt%, 20 µL). Fifteen minutes after the mixture was agitated, the absorbance values at 220 and 275 nm were measured by UV-Vis spectrophotometry. The total NO_3_^−^ absorbance was calculated using the expression [Abs]_tot_ = [Abs]_220 nm_ − 2 × [Abs]_275 nm_, where [Abs]_220 nm_ and [Abs]_275 nm_ represents the absorbance values at 220 and 275 nm, respectively.^[2]^ The total absorbance values were quantitatively compared with the calibration results (Figure S15c) to determine the concentration of the NO_3_^−^ reactant.

**Detection of NH_2_OH**

The NH_2_OH concentration was determined using a colorimetric method based on UV-Vis spectrophotometry.^[3]^ To that end, the reacted electrolyte (1 mL) was sequentially mixed with phosphate buffer (pH 6.8, 1 mL, 0.05 M), trichloroacetic acid (0.2 mL), 8-quinolinol (1 mL), Na_2_CO_3_ (1 mL, 1 M), and deionized water (0.8 mL). The mixture was heated at 35 °C for 1 min in a water bath to induce color formation. Subsequently, the solution was removed from the bath and cooled at room temperature for 15 min. The absorbance at 705 nm was measured and then quantitatively compared with the calibration results (Figure S15d) to determine the concentration of the NH_2_OH byproduct.

**Faradaic efficiency and current density**

The FEs of NH_3_ and NO_2_^−^ production were calculated using the expressions

$FE\left( {NH}_{3} \right)$ = $\frac{(8 \times C_{{NH}_{3}} F \times V)}{Q} \times100 (\%)$ and

$FE\left( {NO}_{2}^{-} \right)$*=* $\frac{(2 \times C_{{NO}_{2}^{-}} F V)}{Q} \times100 (\%)$,

whereas the total current density and partial current density for each substance were calculated as follows:

Total current density = $\frac{Q \times1000}{t \times A}$ ($mA {cm}^{-2}$),

${NH}_{3}$ Partial current density = $FE\left( {NH}_{3} \right)\times\frac{Q \times1000}{t \times A}$ ($mA {cm}^{-2}$), and

${NO}_{2}^{-}$ Partial current density = $FE\left( {NO}_{2}^{-} \right)\times\frac{Q \times1000}{t \times A}$ ($mA {cm}^{-2}$),

where $C_{{NH}_{3}}$ and $C_{{NO}_{2}^{-}}$ are the concentrations (mol L^−1^) of NH_3_ and NO_2_^−^, respectively, as determined by UV-Vis spectrophotometry; *F* is the Faraday constant (96485 C mol^−1^); *V* is the volume of the reacting electrolyte in the working cell (55 mL); *Q* is the total charge consumed at the electrode; *t* is the reaction time (s), and A is the geometric area (cm^2^) of the working electrode measured using an image analysis program (imageJ).

**Measurement of ECSA**

The electrochemical surface area (ECSA) was measured to normalize the NO_3_RR performance to the effective catalytic area participating in the reactions (Figure S17). The ECSAs of the Co-Cu SCC, Co SCC, and Cu SCC were evaluated by conducting cyclic voltammetry (CV) within non-faradaic potential windows at scan rates of 10, 20, 30, 40, and 50 mV s^−1^ (Figures S17a–c). The electrochemical double-layer capacitance (C_dl_) of each catalyst was derived from the slope of the linear plot between the capacitive current density and scan rate (Figure S17d). The C_dl_ values for the Co-Cu SCC, Co SCC, and Cu SCC were found to be 8.76, 7.52, and 7.85 mF cm^−2^, respectively. The ECSAs were subsequently calculated by dividing the C_dl_ values by the specific capacitance (C_s_) of the carbon paper, which was previously estimated to be 0.037 mF cm^−2^.^[4]^ The Co-Cu SCC, Co SCC, and Cu SCC exhibited ECSA values of 236.8, 203.2, and 212.3 cm^2^, respectively.

**Isotope labeling and NH^4+^ quantification by ^1^H-NMR analysis**

To detect NH_4_^+^ by ^1^H-NMR spectroscopy, the target solution (125 µL), maleic acid (15 mM, 125 µL), and H_2_SO_4_ (50 µL) were combined with dimethyl sulfoxide (DMSO)-d_6_ (750 µL).^[5]^ The mixed solution was analyzed using a ^1^H-NMR spectrometer (600 MHz), which revealed two ^15^NH_4_^+^ peaks and/or three ^14^NH_4_^+^ peaks, along with the maleic acid peak of the internal standard. For isotope labeling, the electrolyte that reacted with the 0.1 M K^15^NO_3_ reactant for 1 h at −0.4 V *vs.* RHE was compared with that analyzed by ^1^H-NMR spectroscopy performed using 3 mM ^15^NH_4_Cl and ^14^NH_4_Cl solutions as reference materials. Only the two peaks corresponding to ^15^NH_4_^+^ were detected, confirming that the nitrogen source of the synthesized NH_4_^+^ was solely derived from the NO_3_^−^ reactant. The NH_3_ yield was quantified by normalizing the integral of the NH_4_^+^ peak area, which was proportional to the NH_4_^+^ concentration, relative to the maleic acid internal standard. Calibration of the NH_4_^+^ concentration was performed using standard ^14^NH_4_Cl solutions (1, 2, 3, and 4 mM). The yield of ^14^NH_3_ produced using 0.1 M K^14^NO_3_ as the reactant at −0.4 V *vs.* RHE for 1 h was measured by ^1^H-NMR analysis and UV-Vis spectroscopy to verify the reliability of the colorimetric method.

**Operation of the BPM-based MEA system**

The long-term NO_3_RR was conducted in a custom-built 5 cm^2^ MEA cell with a serpentine flow channel setup that was separated by a bipolar exchange membrane (Fumaesep FME). The cathode—microporous-layer-free carbon paper loaded with the Co-Cu SCC—was fabricated using a spray coating machine for uniform deposition over a large area. A commercial DSA was used as the anode. To ensure uniform contact between the electrodes and the exchange membrane, the cell was assembled using a torque wrench to create equal torque. At the cathode, where the NO_3_RR occurred, a solution of 2 M KNO_3_ and 1 M KOH (50 mL) was circulated at 20 mL min^−1^ from the electrolyte reservoir, while a 1 M KOH solution (50 mL) was circulated on the anode side. A fully sealed circulation system was employed to prevent ammonia volatilization. Reaction electrolyte samples were extracted at 2 h intervals using a syringe to calculate the NH_3_ yield and FE. To convert the produced ammonia into NH_4_Cl salt, the reaction electrolyte was placed in a sealed bottle that was connected to another sealed bottle containing a HCl solution (0.3 M, 200 mL), and inert argon gas (100 sccm) was supplied for 24 h. NH_3_ gas, which was selectively extracted by air stripping, was trapped in the HCl solution to form NH_4_Cl_(aq)_. The removal of water from the product using a rotary evaporator yielded NH_4_Cl salt in the form of a white powder. Residual moisture was removed in a box oven to complete the conversion of NO_3_^−^ to NH_4_Cl_(s)_.

**
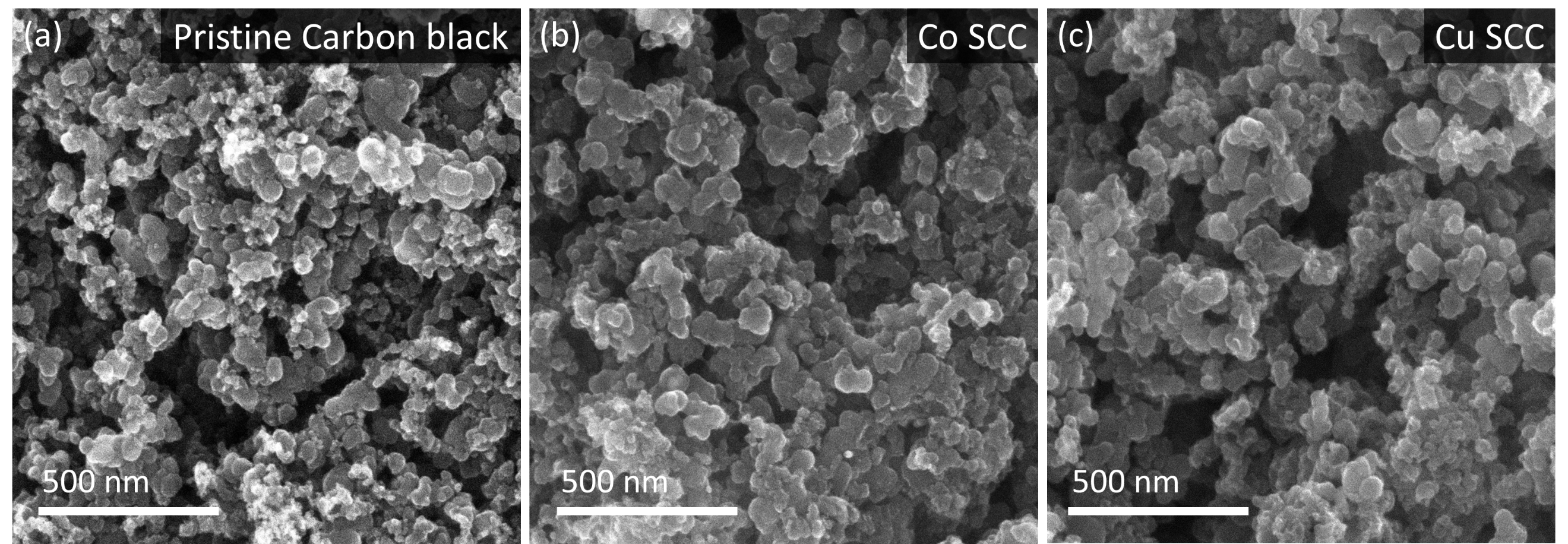
**

**Figure S1.** SEM images of a) bare carbon black, b) the Co SCC, and c) the Cu SCC.


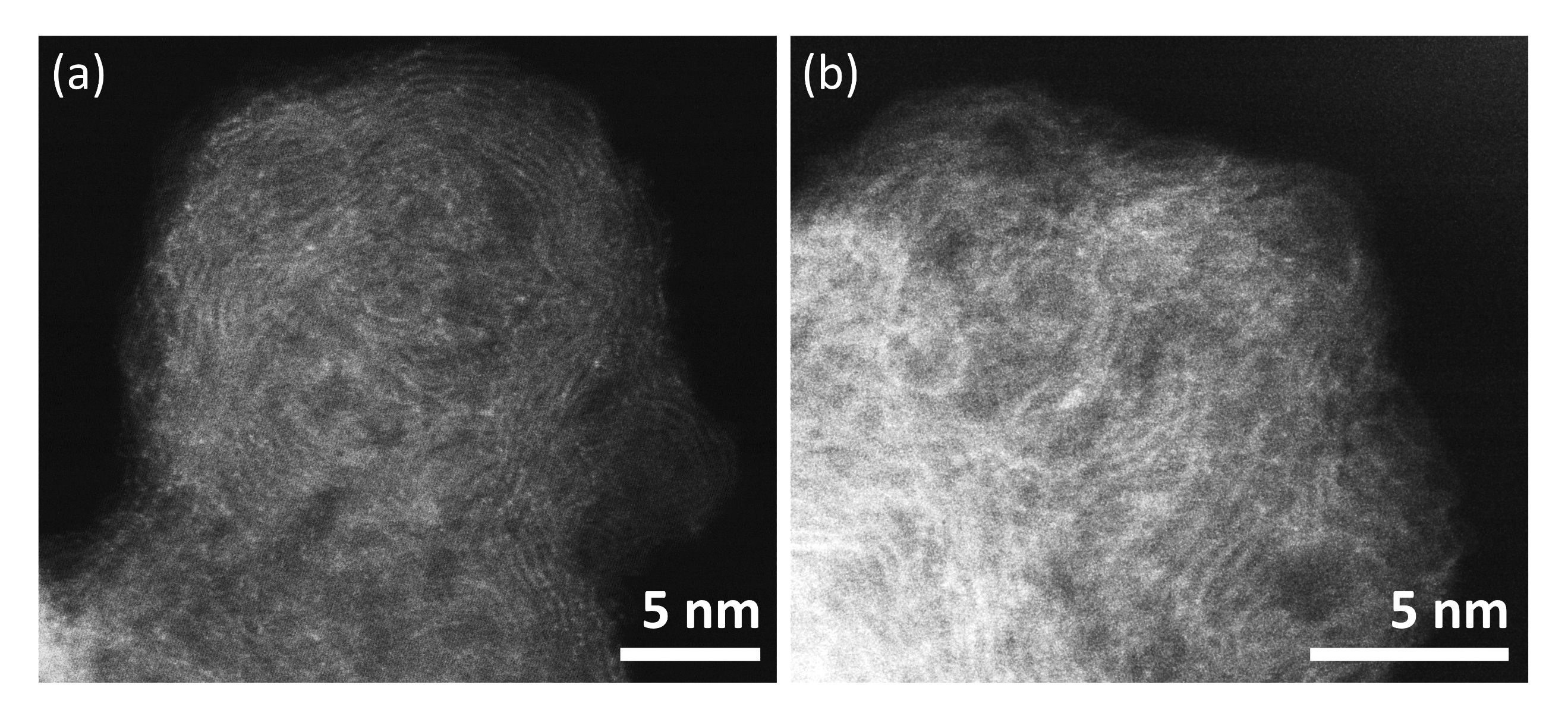
**Figure S2.** High-resolution AC-HAADF-STEM images of the a) Co SCC and b) Cu SCC, indicating the formation of well-dispersed Co or Cu atomic sites on the carbon layer surrounding the carbon black.


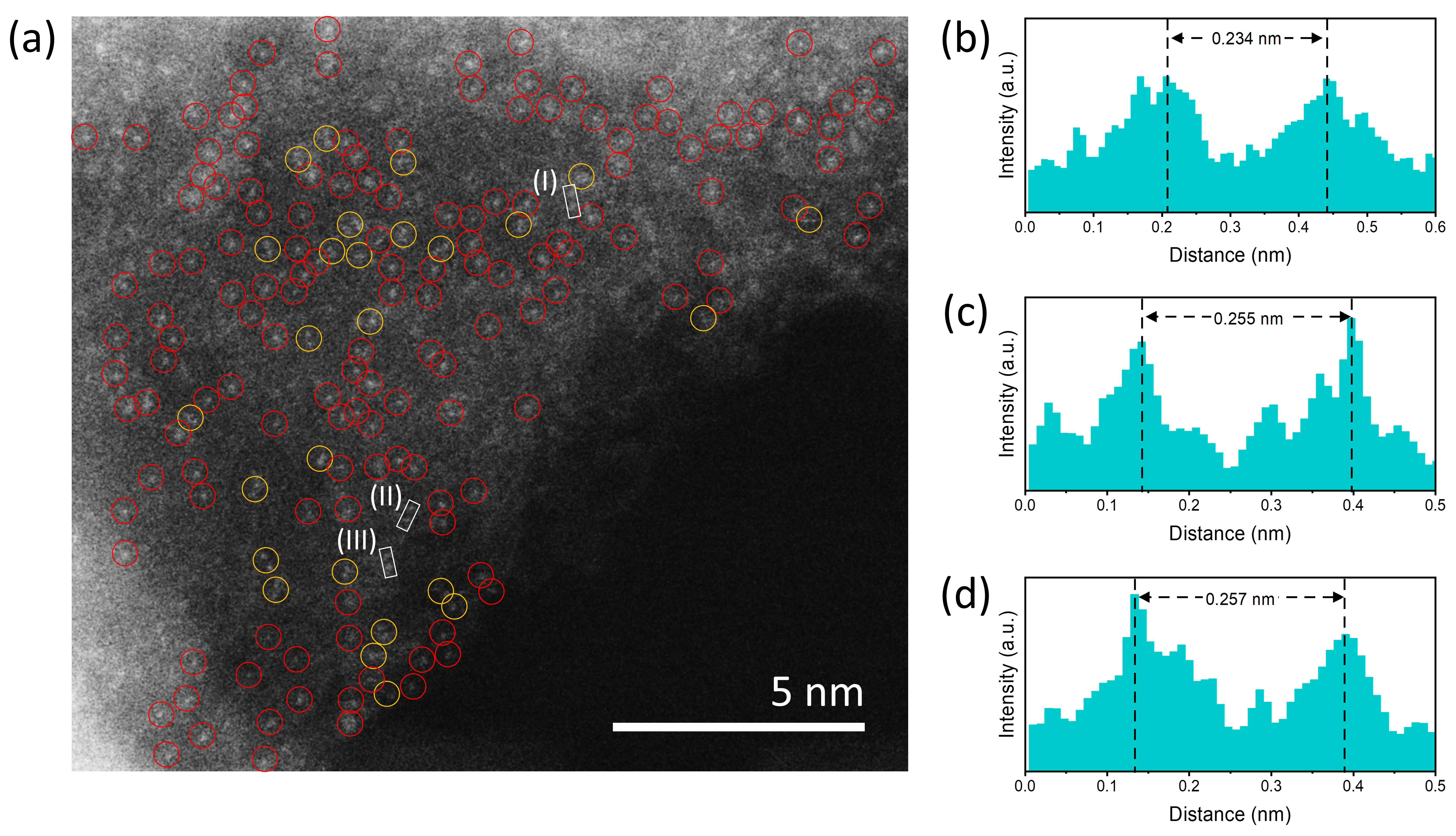


**Figure S3.** (a) AC-HAADF-STEM image of Co-Cu mixed single-atom/cluster (Co-Cu SCC) highlighting atomic active sites (red circles) and diatomic pairs (yellow circles). (b-d) Line-scanning based atomic intensity profiles obtained from regions (I), (II), and (III), respectively, in panel (a).

**Figure S4.** Statistical distribution of the spacing between the closest atomic active sites and the average inter-site distance (*d_site_*) in the Co-Cu SCC.


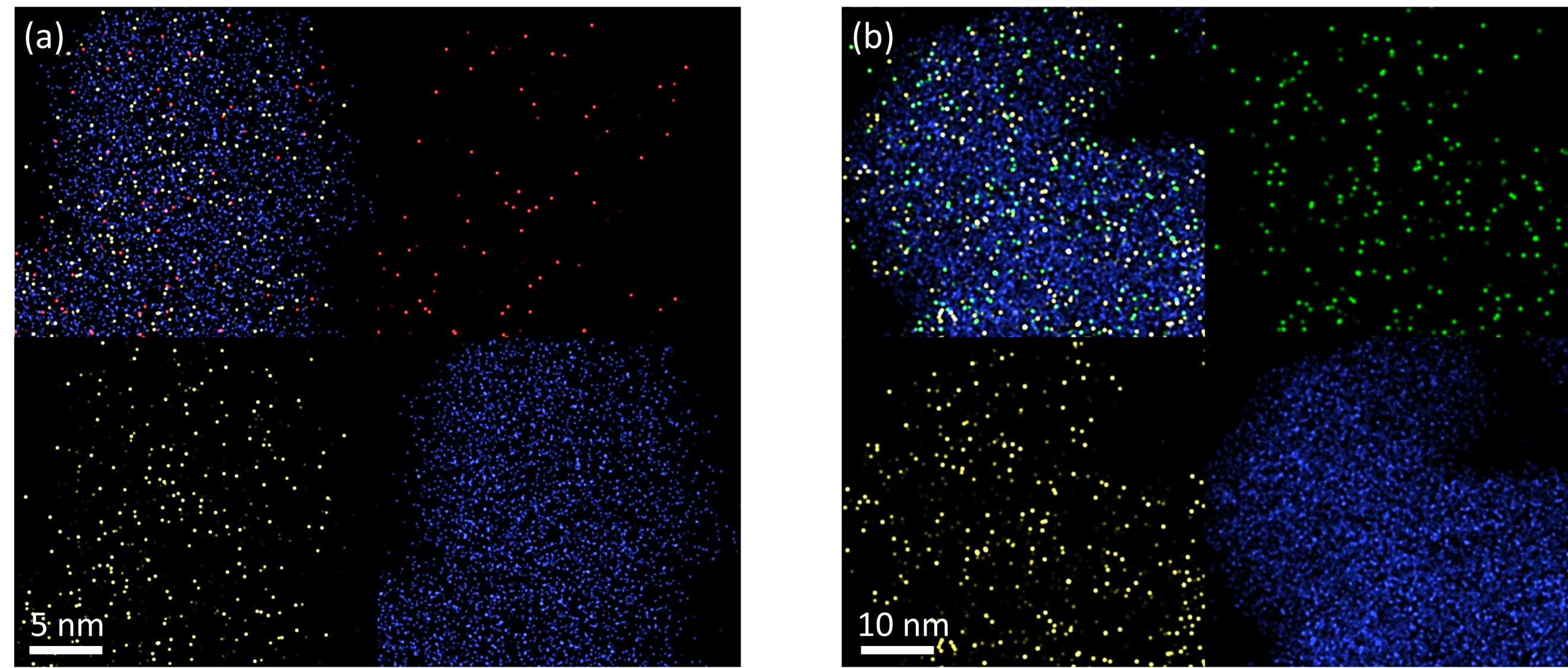
**Figure S5.** EDS-derived maps of Co, N, and C in the a) Co SCC, and those of Cu, N, and C in the b) Cu SCC.

**Figure S6.** O 1s XPS profile of Co-Cu SCC.

**Figure S7.** Cu LMM Auger spectrum of the Co-Cu SCC.


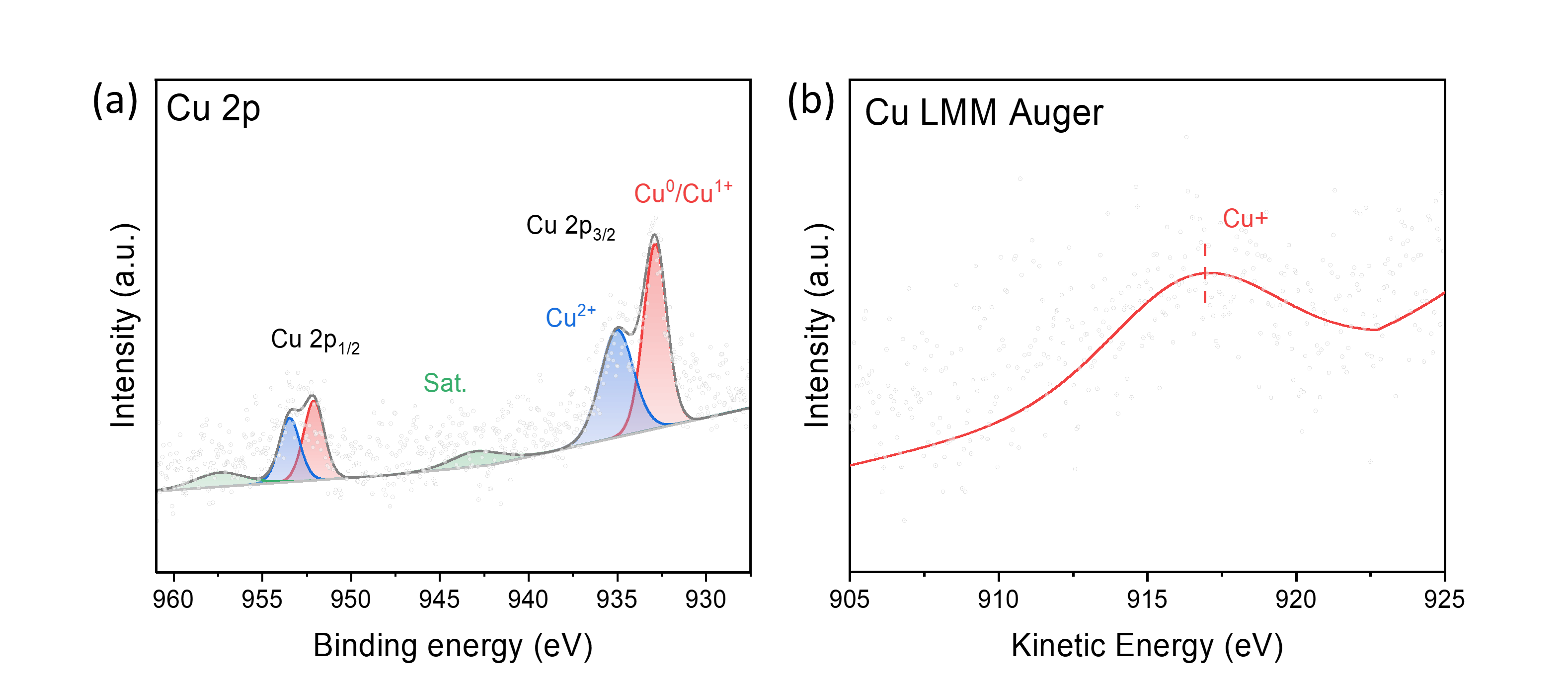


**Figure S8.** a) Deconvoluted Cu 2p XPS profile and b) Cu LMM Auger spectrum of the Co-Cu SCC sample after ion beam etching using Ar sputtering.

**
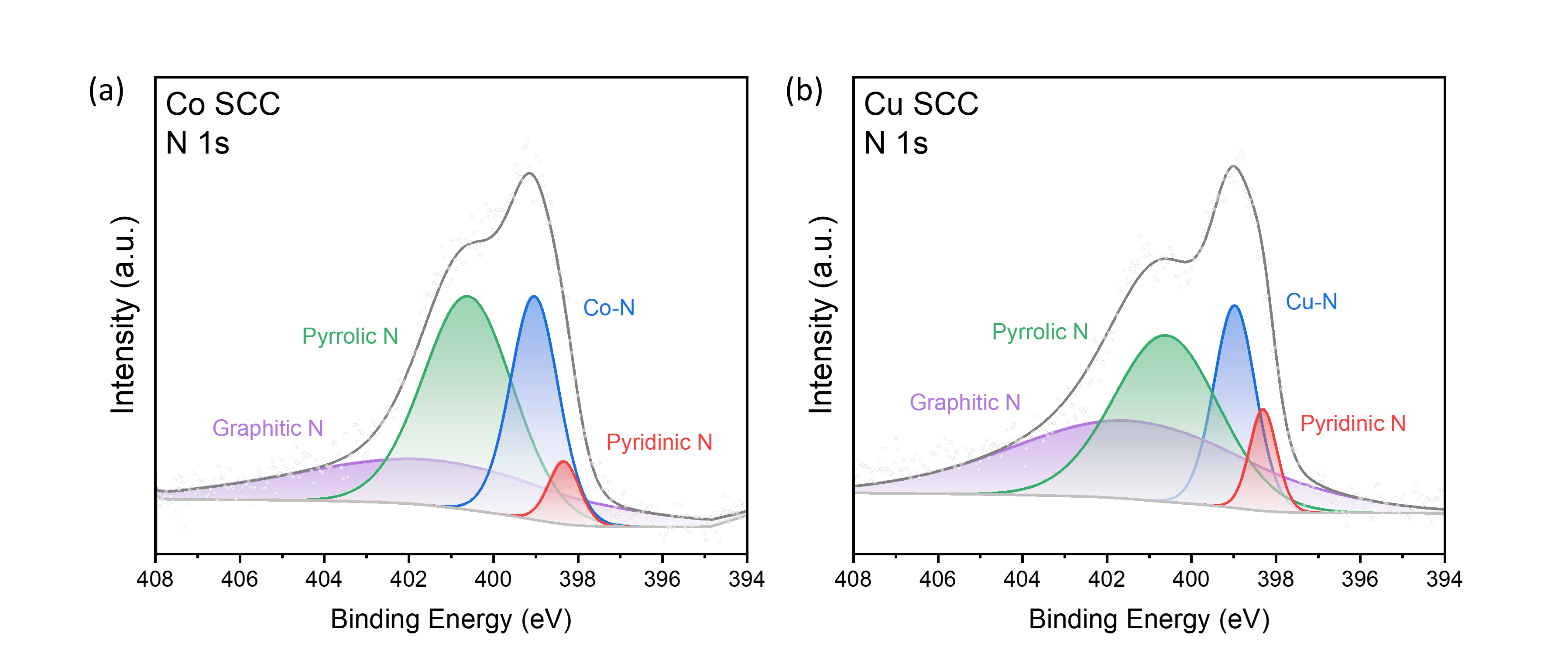
Figure S9.** N 1s XPS profiles of the a) Co SCC and b) Cu SCC.

**
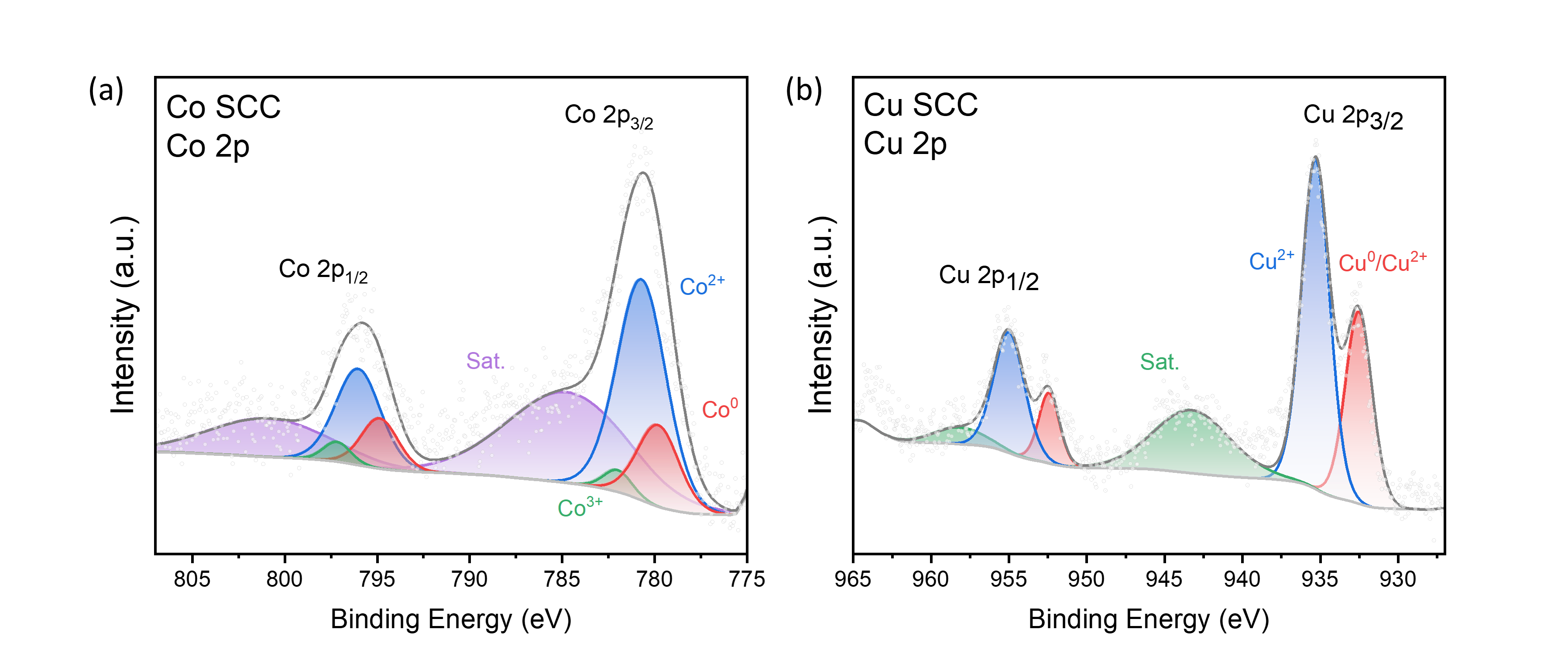
Figure S10.** a) Co 2p XPS profile of the Co SCC, and b) Cu 2p XPS profile of the Cu SCC.

**
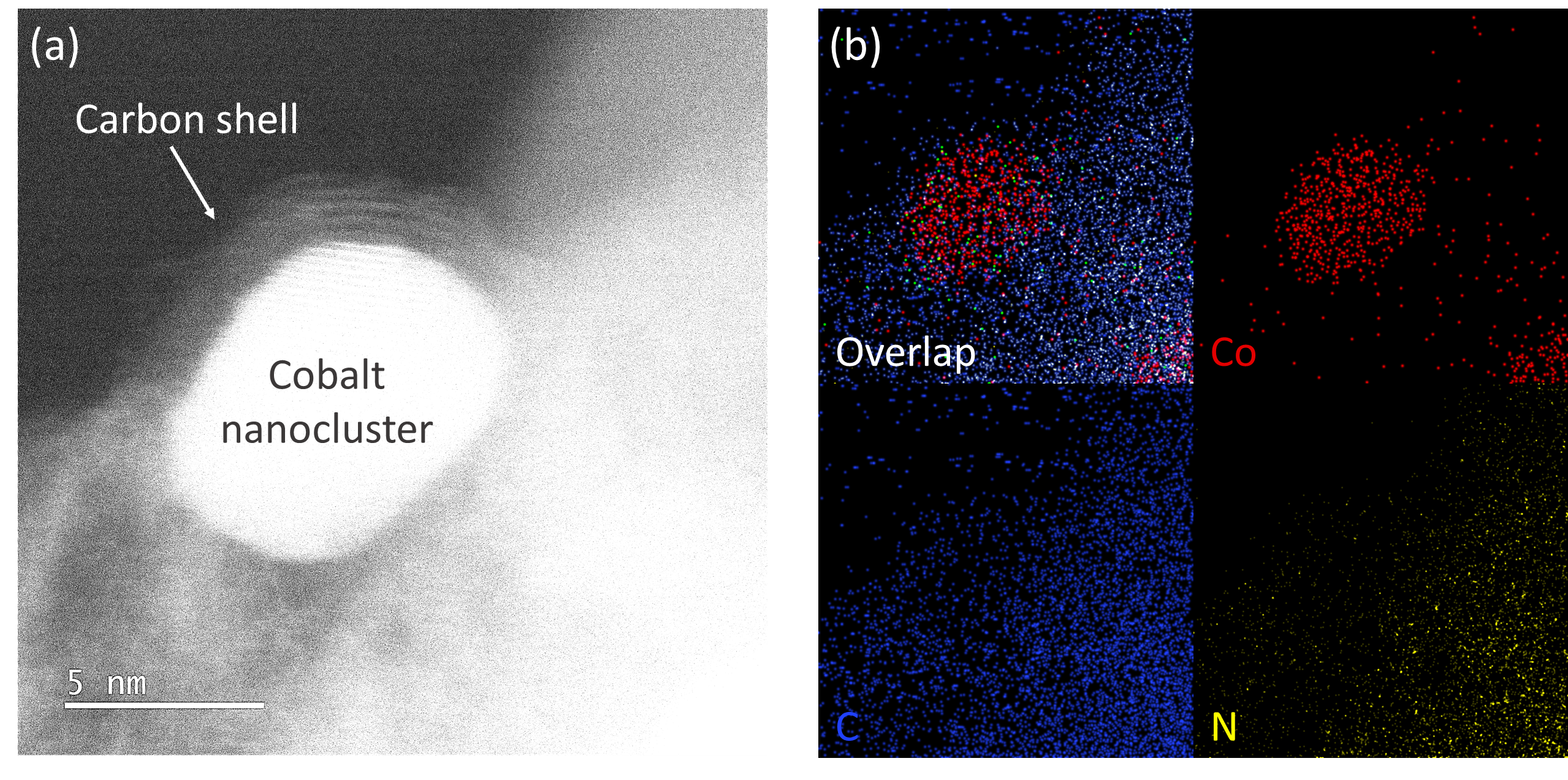
Figure S11.** a) AC-HAADF-STEM image and b) EDS-derived maps of the residual Co nanocluster and the multiple encapsulating carbon shells in the Co-Cu SCC.

**Figure S12.** High-resolution powder XRD patterns of the Co-Cu SCCs with increasing acid treatment times (2, 4, 6, 8, and 10 hours), indicating that metal clusters remain even after prolonged exposure to acid.


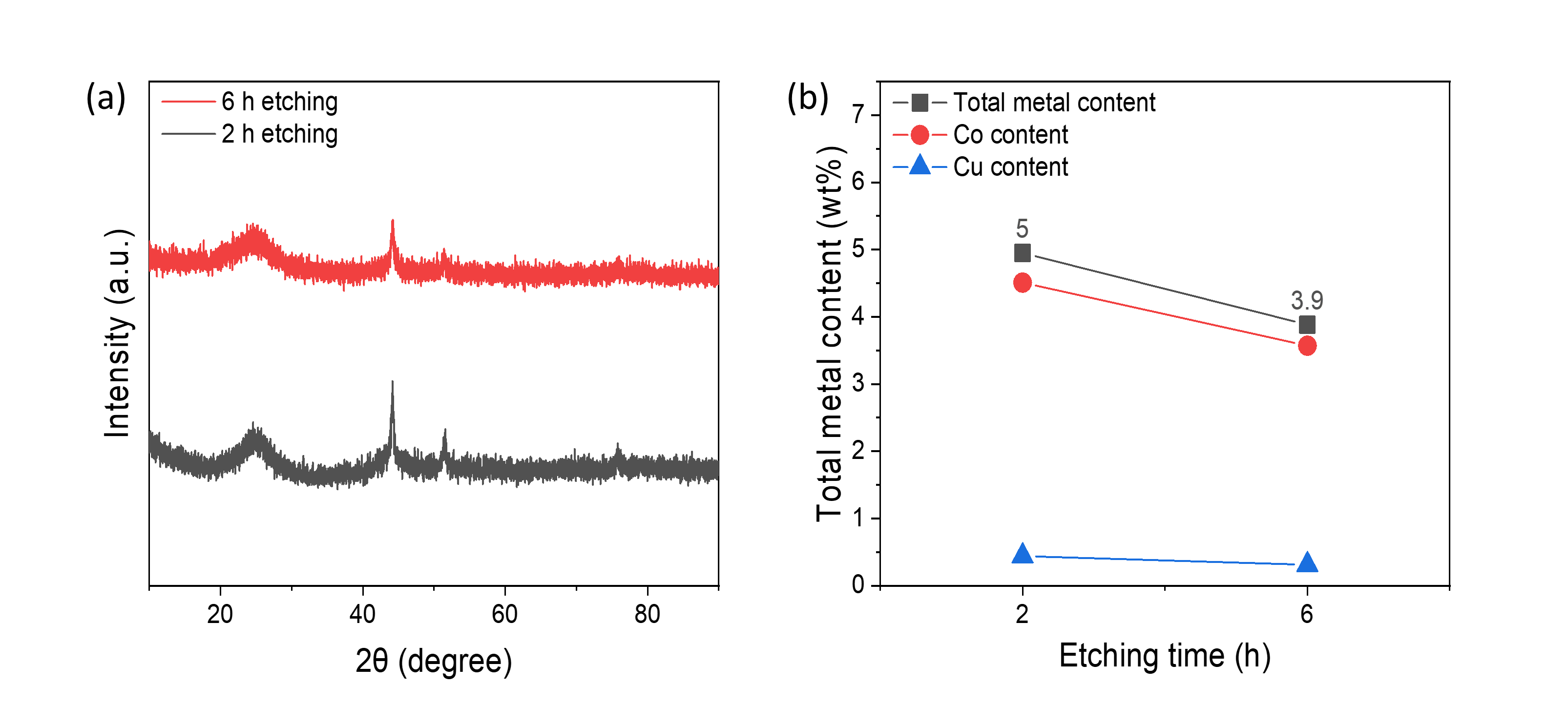


**Figure S13.** a) XRD patterns and b) ICP-OES analysis results of Co-Cu SCCs with different acid etching times (2 and 6 hours). Note that the sample etched for 6 hours is the catalyst discussed in the main text.


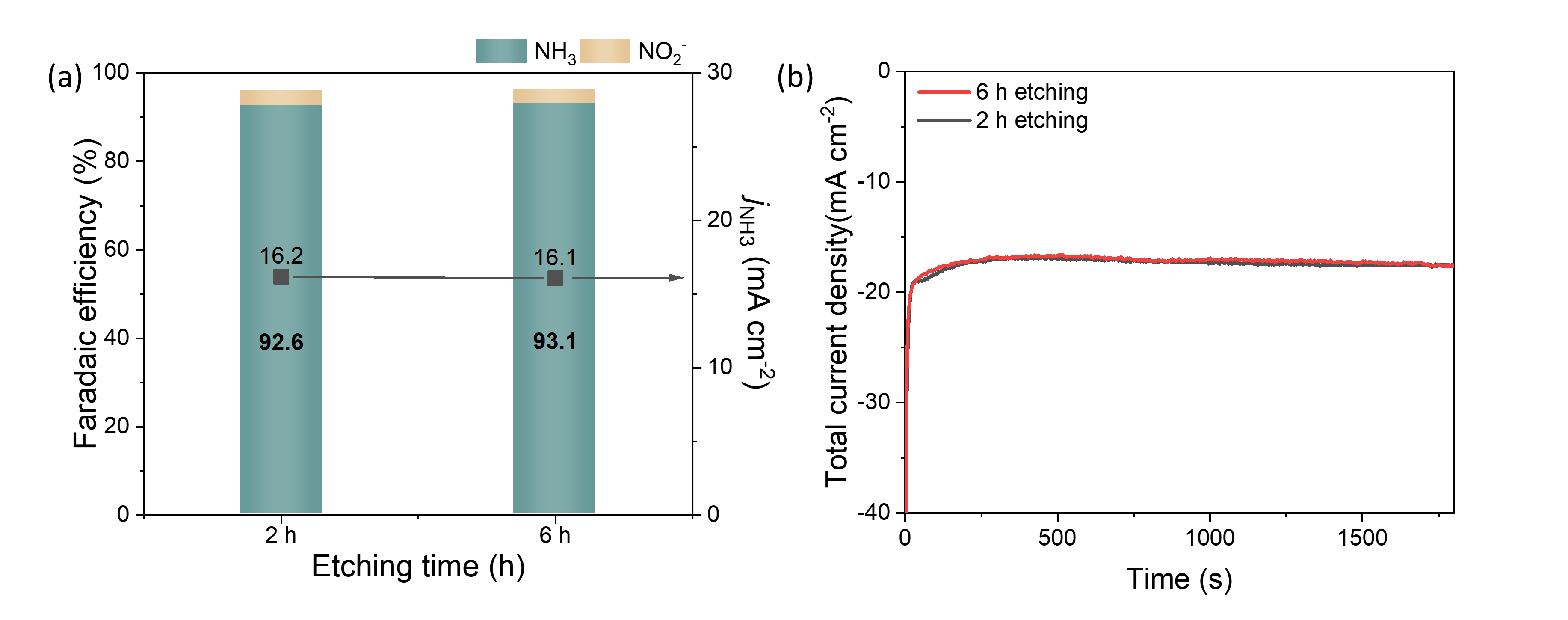


**Figure S14.** Electrochemical NO_3_RR performance results of a) FE, NH_3_ partial current density, and b) total current density of Co-Cu SCCs with different etching times (2 and 6 hours). Note that the sample etched for 6 hours is the catalyst discussed in the main text.

**
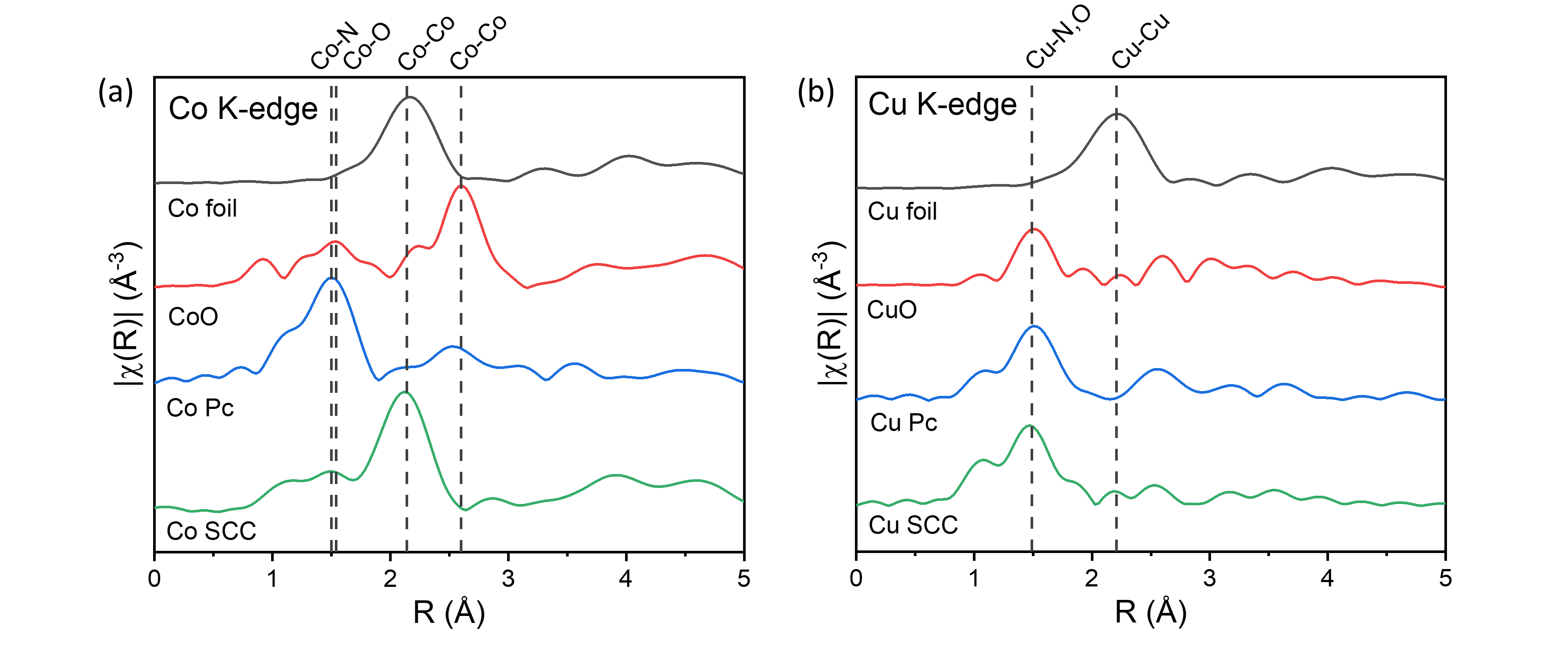
Figure S15.** a) *k*^2^-weighted Co K-edge FT-EXAFS spectra of the Co SCC, Co Pc, CoO, and Co foil. b) *k*^2^-weighted Cu K-edge FT-EXAFS spectra of the Cu SCC, Cu Pc, CuO, and Cu foil.

**
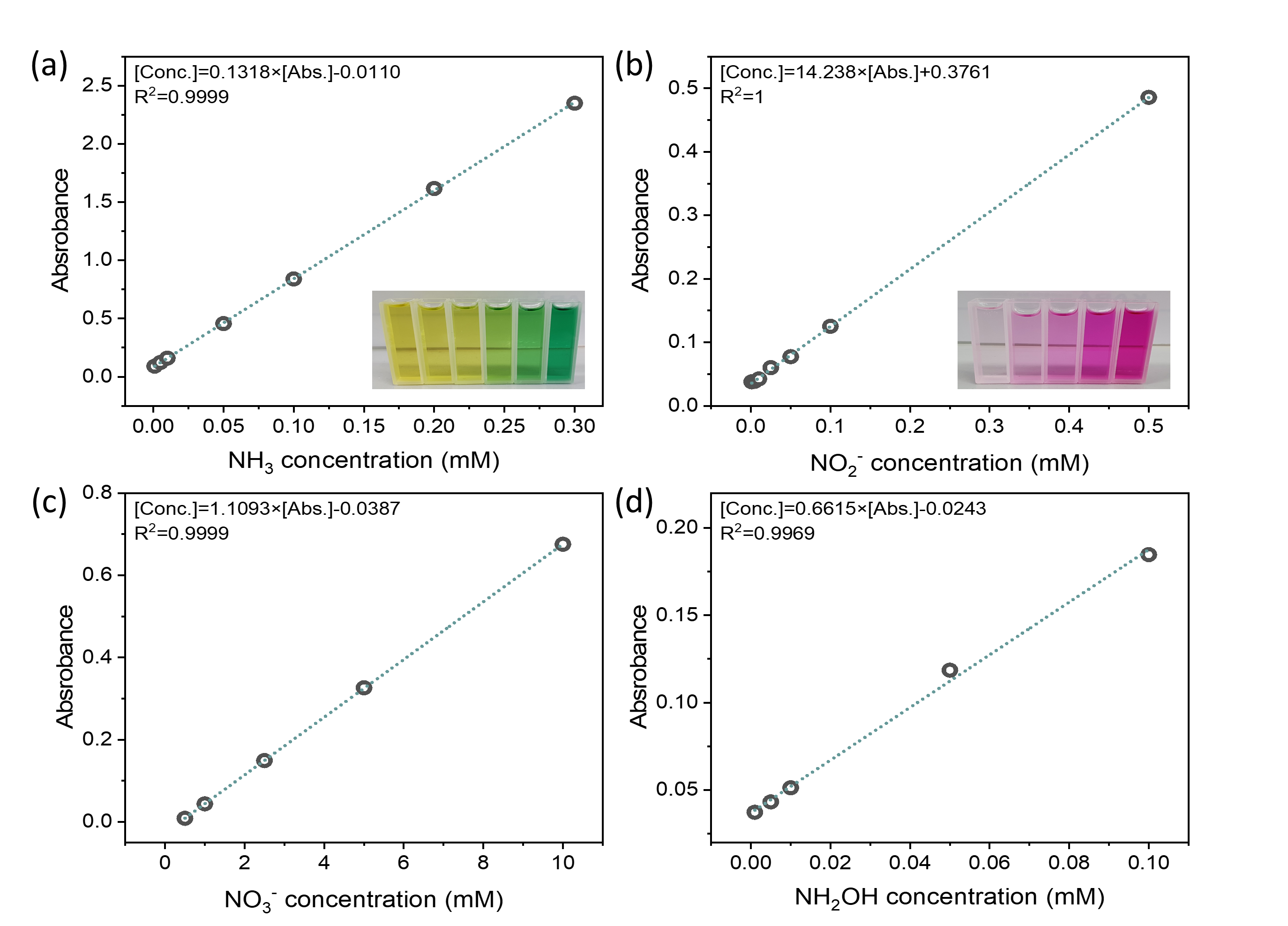
Figure S16.** Concentration–absorbance relationship established by calibration using a colorimetric method for a) NH_3_, b) NO_2_^−^, c) NO_3_^−^, and d) NH_2_OH; inset images in a) and b) show the colors formed by the reagents.

**
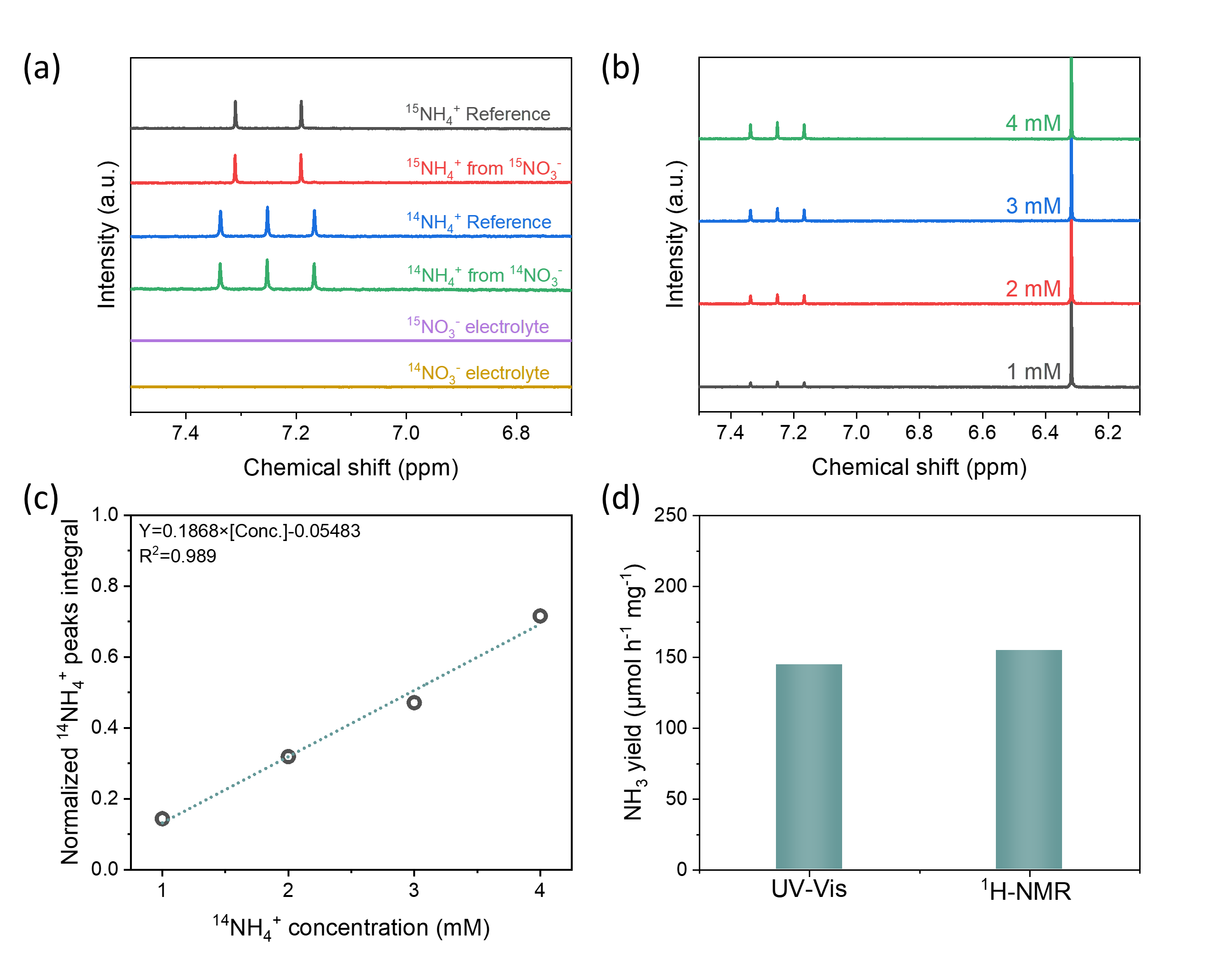
Figure S17.** a) ^1^H-NMR-based isotope labeling analysis of NH_4_^+^ peaks conducted using a 3 mM ^15^NH_4_Cl reference solution, 3 mM ^14^NH_4_Cl reference solution, 1-h-reacted solution with ^14^NO_3_^−^ or ^15^NO_3_^−^ as the reactant, and ^14^NO_3_^−^ and ^15^NO_3_^−^ electrolytes. b) Calibration data and c) linear fit for 1, 2, 3, and 4 mM ^14^NH_4_Cl using maleic acid as the internal standard for ammonia quantification by ^1^H-NMR spectroscopy. d) Comparison of the quantification results obtained by UV–vis and ^1^H-NMR analyses.

**
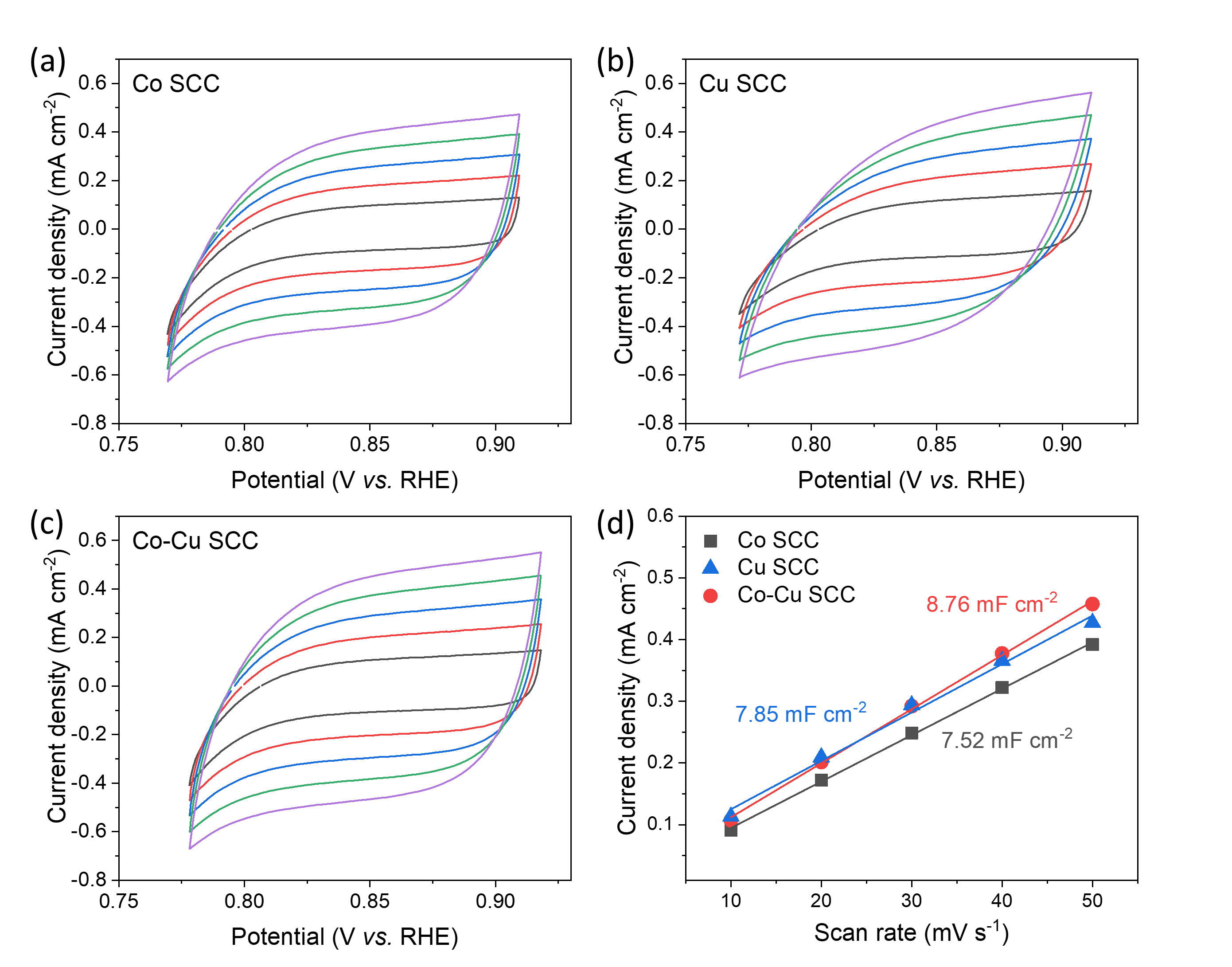
Figure S18.** Cyclic voltammetry (CV) results for the a) Co SCC, b) Cu SCC, and c) Co-Cu SCC at scan rates of 10, 20, 30, 40, and 50 mV s^−1^, and d) linear plots of capacitive current density as a function of the scan rate, along with the electrochemical double-layer capacitance (C_dl_) and ECSA values.

**
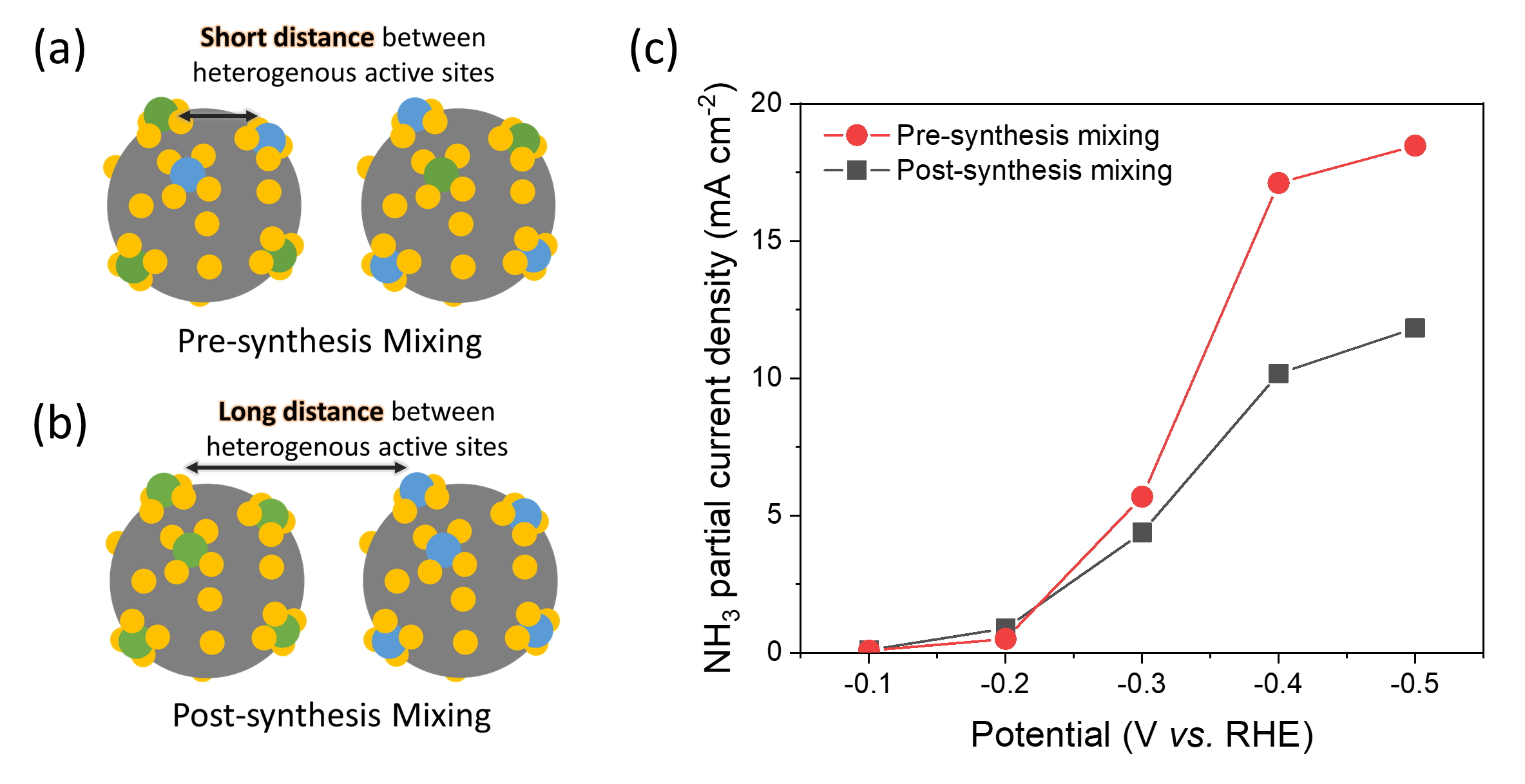
Figure S19.** Schematic illustration of the spacing between heterogeneous active sites in a) pre-synthesis mixing sample (short distance) and b) post-synthesis mixing sample (long distance), and c) graph comparing the NH_3_ partial current density of the two samples, proving that the synergistic effect is enhanced as the spacing shrinks.


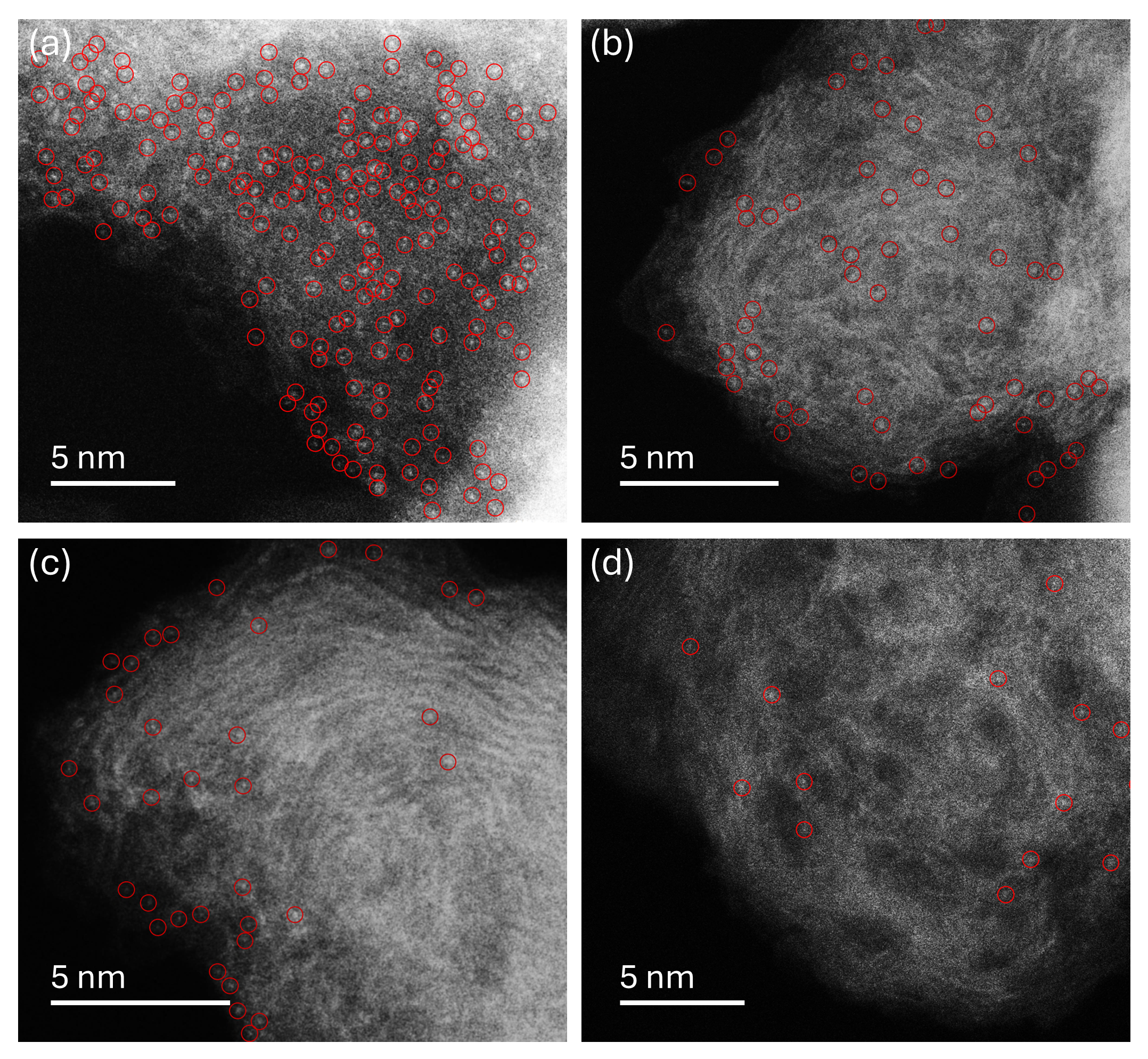


**Figure S20.** AC-HAADF-STEM images of synthesized Co-Cu SCCs with metal contents of a) 3.9 wt%, b) 2.4 wt%, c) 1.2 wt%, and d) 0.82 wt%, demonstrating that, as the metal content decreases, the distance between metal active sites increases.


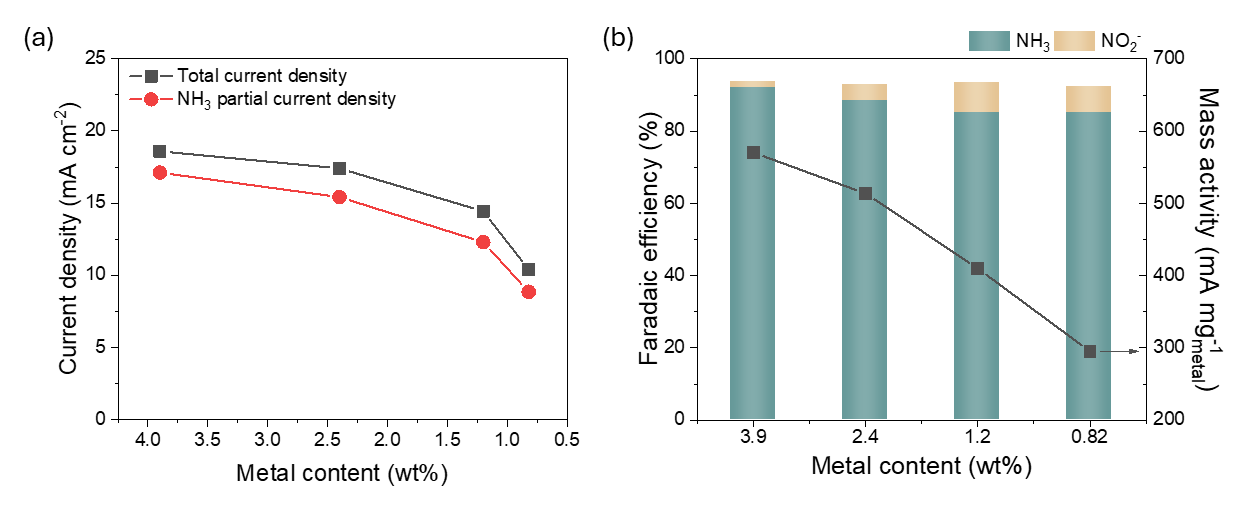


**Figure S21.** Electrocatalytic NO_3_RR performance as a function of the metal content, which is inversely proportional to the interatomic distance between active sites: a) Total current density and NH_3_ partial current density, b) NH_3_ Faradaic efficiency and mass activity.

**Figure S22.** NH_3_ Faradaic efficiency of Co-Cu SCC based on a zero-gap-based MEA in 0.1 M KOH + 1 M KNO_3_ and 1M KOH + 1M KNO_3_ as the electrolytes.


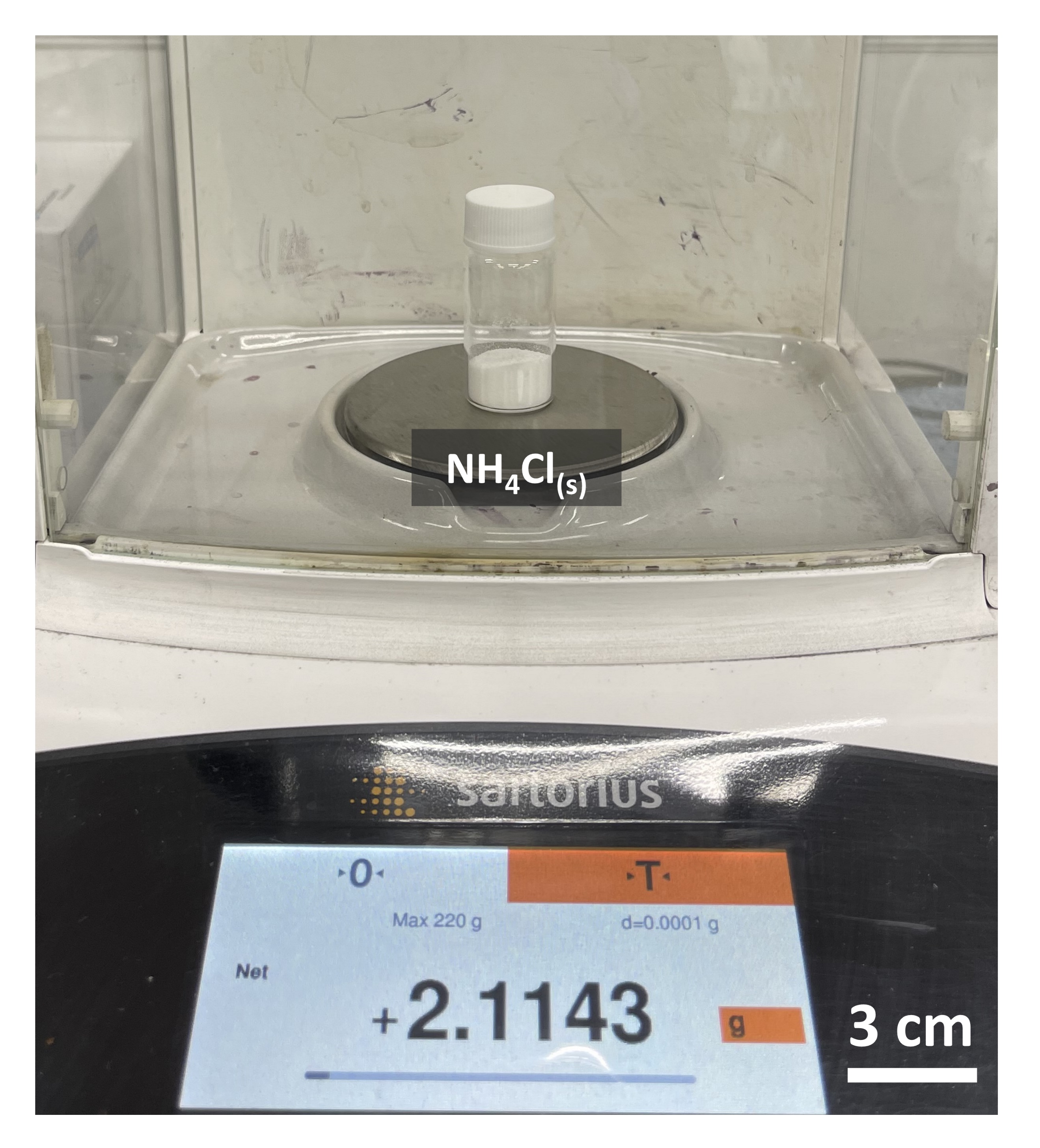
**Figure S23.** Weight measurement of NH_4_Cl salt (2.1 g) produced by operating a BPM-based MEA system integrated with the Co-Cu SCC for 24 h.

**Table S1.** BET surface areas for carbon black, the Co-Cu SCC, Co SCC, and Cu SCC.

| Sample | BET surface area (m^2^ g^–1^) |
| --- | --- |
| Carbon black | 231.74 |
| Co-Cu SCC | 210.08 |
| Co SCC | 201.19 |
| Cu SCC | 153.28 |

**Table S2.** Weight percent of metal elements (Co and Cu) in the Co-Cu SCC, Co SCC, and Cu SCC, as determined from ICP-MS analysis.

|  | Weight percent (%) | |
| --- | --- | --- |
| Sample | Co | Cu |
| Co-Cu SCC | 3.57 | 0.34 |
| Co SCC | 3.99 | – |
| Cu SCC | – | 1.20 |

**Table S3.** Atomic and weight percentages of metal elements (Co and Cu) in the Co-Cu SCC, Co SCC, and Cu SCC, as determined from XPS analysis.

|  | Atomic percent (%) | | Weight percent (%) | |
| --- | --- | --- | --- | --- |
| Sample | Co | Cu | Co | Cu |
| Co-Cu SCC | 0.7 | 0.1 | 3.24 | 0.5 |
| Co SCC | 0.55 | – | 2.58 | – |
| Cu SCC | – | 0.43 | – | 2.2 |

References

[1] Z.-Y. Wu, M. Karamad, X. Yong, Q. Huang, D. A. Cullen, P. Zhu, C. Xia, Q. Xiao, M. Shakouri, F.-Y. Chen, J. Y. Kim, Y. Xia, K. Heck, Y. Hu, M. S. Wong, Q. Li, I. Gates, S. Siahrostami, H. Wang, *Nat. Commun.* **2021**, *12*, 2870.

[2] Z. Gong, W. Zhong, Z. He, Q. Liu, H. Chen, D. Zhou, N. Zhang, X. Kang, Y. Chen, *Appl. Catal. B-Environ.* **2022**, *305*, 121021.

[3] D. S. Frear, R. C. Burrell, *Anal. Chem.* **1955**, *27*, 1664-1665.

[4] G. Hwa Jeong, Y. Chuan Tan, J. Tae Song, G.-Y. Lee, H. Jin Lee, J. Lim, H. Young Jeong, S. Won, J. Oh, S. Ouk Kim, *Chem. Eng. J.* **2021**, *426*, 131063.

[5] W. He, J. Zhang, S. Dieckhöfer, S. Varhade, A. C. Brix, A. Lielpetere, S. Seisel, J. R. C. Junqueira, W. Schuhmann, *Nat. Commun.* **2022**, *13*, 1129.
